# Ancient DNA and osteological analyses of a unique paleo-archive reveal Early Holocene faunal expansion into the Scandinavian Arctic

**DOI:** 10.1126/sciadv.adk3032

**Published:** 2024-03-29

**Authors:** Aurélie Boilard, Samuel J. Walker, Trond Klungseth Lødøen, Mona Henriksen, Liselotte M. Takken Beijersbergen, Bastiaan Star, Marius Robu, Christine Tøssebro, Cornelia Marie Albrektsen, Yvonne Soleng, Sverre Aksnes, Roger Jørgensen, Anne Karin Hufthammer, Thijs van Kolfschoten, Stein-Erik Lauritzen, Sanne Boessenkool

**Affiliations:** ^1^ Centre for Ecological and Evolutionary Synthesis (CEES), Department of Biosciences, University of Oslo, Oslo, Norway.; ^2^ Department of Cultural History, University Museum of Bergen, University of Bergen, Bergen, Norway.; ^3^ Faculty of Environmental Sciences and Natural Resource Management, Norwegian University of Life Sciences, Ås, Norway.; ^4^ Department of Natural History, University Museum of Bergen, University of Bergen, Bergen, Norway.; ^5^ Department of Karstonomy, Karst Inventory and Protection, Emil Racoviţă Institute of Speleology, Bucharest, Romania.; ^6^ Department of Earth Sciences, University of Bergen, Bergen, Norway.; ^7^ The Arctic University Museum of Norway, University of Tromsø, Tromsø, Norway.; ^8^ Faculty of Archaeology, Leiden University, Leiden, Netherlands.; ^9^ Joint International Research Laboratory of Environment and Social Archaeology, Shandong University, Qingdao, China.; ^10^ Department of Earth Science, Bjerknes Centre for Climate Research, University of Bergen, Bergen, Norway.; ^11^ Centre for Early Sapiens Behaviour (SapienCE), University of Bergen, Bergen, Norway.

## Abstract

Paleo-archives are essential for our understanding of species responses to climate warming, yet such archives are extremely rare in the Arctic. Here, we combine morphological analyses and bulk-bone metabarcoding to investigate a unique chronology of bone deposits sealed in the high-latitude Storsteinhola cave system (68°50′ N 16°22′ E) in Norway. This deposit dates to a period of climate warming from the end of the Late Glacial [~13 thousand calibrated years before the present (ka cal B.P.)] to the Holocene thermal maximum (~5.6 ka cal B.P.). Paleogenetic analyses allow us to exploit the 1000s of morphologically unidentifiable bone fragments resulting in a high-resolution sequence with 40 different taxa, including species not previously found here. Our record reveals borealization in both the marine and terrestrial environments above the Arctic Circle as a naturally recurring phenomenon in past periods of warming, providing fundamental insights into the ecosystem-wide responses that are ongoing today.

## Teaser

A unique Early Holocene cave deposit reveals postglacial faunal expansion into the Scandinavian Arctic.

## INTRODUCTION

Global warming is occurring at an unprecedented pace (*1*), with high-latitude regions warming at an accelerated rate compared to others (*2*). This warming is associated with observations of poleward shifts in the distribution of species in both marine and terrestrial environments (*3*–*6*), although it remains unclear how resilient many species are in adapting to and tracking shifting habitats (*3*, *7*). Past periods with rapid climate change can be an analog for current and future change [see, e.g., (*8*–*10*)] and paleo-archives from such periods often provide the only opportunity to observe species and community turnover following previous dramatic climatic and environmental transitions [see, e.g., (*11*, *12*)]. These archives are therefore essential to help improve predictions of species’ resilience and the response of ecosystems and biodiversity today (*13*–*16*), especially in those areas with the fastest rates of change.

Fennoscandia experienced extreme climatic and environmental alterations following the retreat of the Scandinavian Ice Sheet during the Late Glacial period [~14.6–11.7 thousand years (ka) ago] well into the Early Holocene (11.7–8.2 ka). This glacial retreat was followed by a prolonged period of warming known as the Holocene Thermal Maximum (HTM; ~7.5–5.5 ka), with local temperatures being warmer than today (*17*–*19*), which provides a valuable analog for current and near-future climate change (*8*). However, we lack a thorough understanding of faunal distributions in Fennoscandia during these periods. This lack of understanding is caused in part by the fact that the sequence of ice retreat was complex, making it difficult to reconstruct local climatic conditions. For instance, the retreat of ice was interrupted by standstills and even periodically reverted during short cold periods such as the Younger Dryas stadial (~12.9–11.7 ka), before a final disintegration of the glaciers during the Early Holocene around 9 ka (*20*). Moreover, there exist few identifiable faunal remains from high latitudes that date to the Late Glacial–Early Holocene transition (*21*–*24*). This rareness is caused by a lack of systematic sieving during earlier excavations, combined with poor soil conditions for the preservation of bone and high levels of fragmentation that make morphological identification difficult or impossible (*25*). Combined, these factors have prevented a comprehensive overview of past fauna during this period of extreme environmental turnover in high-latitude Fennoscandia.

The few identified faunal remains from Scandinavia covering the Late Glacial–Holocene period show that pioneer subarctic and tundra species (e.g., *Lepus timidus* and *Rangifer tarandus*) returned soon after the ice sheet retreated (*24*, *26*). Such pioneers were followed by multiple migrations of warmer-adapted species throughout the Holocene as the climate and environment changed (*24*, *27*). Routes of colonization appear to have been diverse, with evidence for dispersal from the south (i.e., through Denmark) and from the northeast [i.e., the Kola peninsula; (*23*, *28*, *29*)]. Moreover, fossil sequences from Denmark reveal differences in species response times, fluctuations in biodiversity, and periods of overlap in faunal communities (*24*). Nonetheless, insights from such faunal remains are either methodically limited, depending on the sparse number of bones that could be morphologically identified, or geographically restrained, being located in southern or mid-Fennoscandia. These limitations are particularly evident in Norway where early sites such as Skjonghelleren (*30*), Vistehola (*31*, *32*), and Skipshelleren (*33*, *34*) are located in the south apart from Sirijorda (*28*) which is just below the Arctic Circle. No faunal sequences exist from above the Arctic Circle, and to date, no faunal assemblage has been analyzed from Fennoscandia through a combined osteological and paleogenetic methodology.

**Fig. 1.**
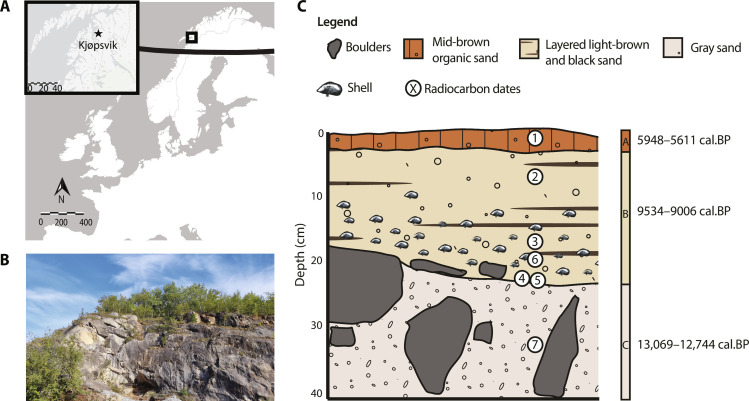
The Nygrotta entrance of the Storsteinhola cave system. (**A**) Map of Fennoscandia and surrounding countries, showing the location of the Storsteinhola cave system in Kjøpsvik village in the municipality of Narvik, Nordland, Norway. The black line represents the Arctic Circle. (**B**) Photograph of the Nygrotta entrance (photo credit: T.K.L.). (**C**) Stratigraphic record of Nygrotta showing the excavated sediment deposits; layer A is a mid-brown organic sand forming the current cave floor, layer B is a sand deposit consisting of interbedded light brown and black bands from weathered marble and amphibolite, and layer C is a gray sand deposit with occasional large rounded and angular cobbles. Encircled numbers show the location of the seven radiocarbon samples. Information about the radiocarbon samples and their dates is presented in table S1.

Here, we present a high-resolution assessment of the faunal diversity from Nygrotta, a former entrance of the Storsteinhola cave system in Kjøpsvik, municipality of Narvik, northern Norway (Fig. 1A). Nygrotta was first discovered in 1993 during road construction, removing the scree which had sealed the entrance (*35*). Preliminary investigations in the same year recovered a dense mussel shell layer as well as teeth from small rodents and bones from fish and birds dating to 8285 ± 105 before the present (B.P.) and 5805 ± 75 B.P. (*35*, *36*). These preliminary investigations evidenced the uniqueness of Nygrotta as a coastal site in Fennoscandia above the Arctic Circle where Early Holocene fauna is preserved and highlighted its potential for analyses of faunal communities following the retreat of the Scandinavian Ice Sheet during the Late Glacial and into the Holocene. By integrating paleogenetic tools with osteological analyses, we maximize the potential for high-resolution tracking of the succession and establishment of fauna following the Late Glacial–Holocene transition and into the HTM. Subfossil remains were identified by combining morphological identification with bulk-bone metabarcoding (BBM), which takes advantage of highly variable sequences of the mitochondrial genome that are used as barcodes to identify multiple bone fragments simultaneously (*37*, *38*). This multi-method approach can detect taxa from otherwise unidentifiable fragments, increasing the faunal representation of a site’s biodiversity [see, e.g., (*39*–*41*)]. We retrieve an exceptional faunal sequence of 40 taxa from a period of dramatic climatic and environmental change and highlight clear shifts in species distributions, including the presence of taxa not previously identified so far north. These results provide evidence for borealization as a naturally recurring phenomenon in the past.

## RESULTS

### A Late Glacial–Holocene transition sequence

Our excavations at Nygrotta intercepted three distinct stratigraphic layers (A, B, and C) representing a sequence ranging from the Late Glacial interstadial–Younger Dryas transition to the Middle Holocene, based on radiocarbon dates of five shells and one bone (Fig. 1, fig. S1, and table S1). Layer C (>20 cm thickness) is a gray, massive sand deposit with occasional large rounded and angular cobbles, dated to 13,069–12,744 calibrated years before the present (cal B.P.). Layer B (25 cm thickness) is a sand deposit with subhorizontal, uneven layers consisting of interbedded light brown and black bands from weathered marble and amphibolite, respectively. Three mechanical layers were excavated in layer B. The lowermost mechanical layer (B3) is characterized by an increased presence of mussel shells, while there are fewer shells above (mechanical layers B1 and B2). Radiocarbon ages of shells and bone from upper (B1) and lower (B3) mechanical layers overlap within the Early Holocene (combined range between 9534–9006 cal B.P.; table S1). Despite the presence of some charcoal in layer B, there are no indications that these are the result of human activity, and rather indicate potential forest fires or that charcoal was brought into the cave from the surroundings by water transport, drainage, or similar natural processes. The uppermost layer A (2- to 5-cm thick) is characterized as a mid-brown massive organic sand, which forms the current cave floor surface; a shell dates the layer to the Middle Holocene (5948–5611 cal B.P.; table S1), which corresponds with the 5805 ± 75 B.P. date obtained during the 1993 investigations (*35*, *36*). No further buildup of sediment occurred after this point as the entrance to the cave was blocked by scree from above, effectively sealing the cave off until its discovery in 1993 during road construction.

### Taphonomy and exploring for archeology

A total number of 2381 bone fragments were recovered during our excavation in 2021. Taphonomic analysis showed a high degree of bone fragmentation but provided no signs of abrasion or rounding as indicators of transport by water. We did not obtain conclusive evidence for corrosion or digestion of the bones caused by predators, and neither did we find any indication of linear marks associated with cutting, as could have been caused by humans. Bone element distribution was dominated by teeth and vertebrae, which are known to be robust elements benefiting long-term preservation (table S2). Some quartz and quartzite pieces with characteristics that could indicate human influence were initially sampled for detailed examination, but following closer inspection in the laboratory, these were not deemed to have been worked by humans and no further evidence to suggest human presence at the site was found.

### Taxonomic identification of faunal remains

**Fig. 2.**
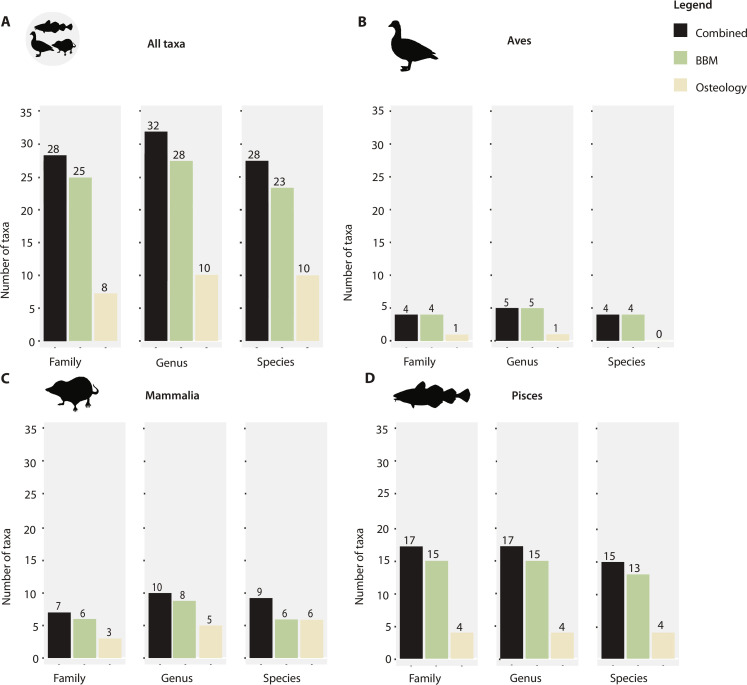
Comparison of the number of identified taxa by BBM and osteology at different taxonomic levels. The number of taxa identified by each method is shown at the family, genus, and species level. The numbers above bars present the number of taxa. (**A**) All taxa (excluding Ranidae); (**B**) Aves; (**C**) Mammalia; (**D**) Pisces.

Osteological analysis identified 161 bone fragments (6.8%; table S3) to at least family level, representing 12 taxa from 8 families, 10 genera, and 10 species (Fig. 2 and Table 1). The remaining 93.2% (2220 fragments) of the bone assemblage was unidentifiable by comparative morphology and sorted into Mammalia (241 fragments), Pisces (1138 fragments), Aves (2 fragments), and unidentifiable vertebrates (839 fragments; table S3). For the ancient DNA (aDNA) BBM analyses, we obtained a total of 50.4 million raw sequence reads (table S4), which following initial filtering and taxonomic assignment resulted in 165,626 fish, 54,764 mammal, and 9386 bird sequence variants. From these variants, we identified 34 taxa, comprising 26 families, 28 genera, and 23 species when requiring a 98% identity match for mammals and 95% for fish and birds (Fig. 2, Table 1, and table S5).

**Table 1.** Taxa identified by BBM and osteology (OST) per stratigraphic layers (A, B, and C) for Nygrotta. A total of 40 taxa were identified by osteological and BBM analyses, representing 29 families, 32 genera, and 28 species. Ages given in calibrated years before present (cal B.P.).

ID	English name	A	B	C
		5948–5611 cal B.P.	9534–9006 cal B.P.	13,069–12,744 cal B.P.
**Aves**				
Phasianidae	Pheasants, grouse, and allies	BBM	-	-
Tetraoninae	Grouse	BBM	-	-
*Lyrurus tetrix*	Black grouse	BBM	-	-
*Tetrao urogallus*	Western capercaillie	BBM	-	-
*Tetrastes bonasia*	Hazel grouse	BBM	-	-
Anatidae	Ducks, geese, and waterfowl	-	BBM	-
*Anser* sp.	Gray and white geese	-	BBM + OST	-
*Fratercula arctica*	Atlantic puffin	-	BBM	-
Laridae	Gulls	-	BBM	-
**Mammalia**				
*Lepus timidus*	Mountain hare	-	BBM	-
Cricetidae/Arvicolinae	Voles, lemmings, and muskrats	OST	OST	OST
*Microtus* sp.	Voles	OST	OST	OST
*Microtus agrestis*	Short-tailed field vole	BBM + OST	BBM + OST	-
*Alexandromys oeconomus*	Tundra vole	OST	OST	-
*Clethrionomys glareolus*	Bank vole	BBM	BBM + OST	-
*Myodes rutilus*	Northern red-backed vole	BBM	BBM	-
*Lemmus lemmus*	Norwegian lemming	-	BBM	-
*Sorex* sp.	Shrews	BBM	BBM	-
*Sorex araneus*	Common shrew	-	OST	-
*Sorex minutus*	Eurasian pygmy shrew	BBM	OST	-
*Felis* sp.	Wild/domestic cat	BBM	BBM	-
Phocidae	Earless seals	-	BBM	-
*Ursus arctos*	Brown bear	-	OST	-
*Canis* sp.	Wolf/dog	-	BBM	-
**Pisces**				
*Gobio gobio*	Gudgeon	-	BBM	-
*Barbatula* sp.	Stone loaches	-	BBM	-
Salmonidae	Salmon, trout, chars, etc.	-	BBM	-
*Esox lucius*	Northern pike	BBM	-	-
*Molva molva*	Common ling	-	BBM + OST	BBM
*Brosme brosme*	Cusk	-	BBM	-
*Gaidropsarus argentatus*	Arctic rockling	-	BBM	BBM
Gadidae	Cods and haddocks	BBM	BBM + OST	BBM
Gadoidei	Subfamily of Gadidae	BBM	BBM	BBM
*Gadus morhua*	Atlantic cod	BBM	BBM + OST	BBM
*Melanogrammus aeglefinus*	Haddock	-	OST	-
*Pollachius virens*	Saithe	BBM	BBM	-
*Seriola* sp.	Amberjacks	BBM	-	-
Pleuronectidae	Righteye flounders	BBM	BBM	BBM
*Hippoglossus hippoglossus*	Atlantic halibut	-	BBM	-
*Limanda limanda*	Common dab	BBM	BBM	-
Labridae	Wrasses	-	OST	OST
Sebastidae	Rockfish and thornyheads	-	BBM	-
*Eutrigla gurnardus*	Grey gurnard	-	OST	-
Cottidae	Sculpins	BBM	BBM	BBM
*Taurulus bubalis*	Long-spined bullhead	-	BBM	-
Cyclopterinae	Lumpfishes	-	BBM	-
*Cyclopterus lumpus*	Lumpfish	-	BBM	-
*Pholis* sp.	Gunnels	-	BBM	-
*Pholis gunnellus*	Rock gunnel	BBM	BBM	-
Lycodinae	Eelpouts	-	BBM	-
*Anarhichas* sp.	Wolffish	BBM	BBM	-
*Anarhichas lupus*	Atlantic wolffish	BBM	BBM	-
**Amphibia**				
Ranidae	True frogs	-	BBM	-

Consistently more taxa were identified with aDNA BBM compared to comparative morphology at all taxonomic levels, in particular for Pisces (Fig. 2). Combined, the analyses resulted in the identification of 40 taxa representing a total of 29 families, 32 genera, and 28 species (Fig. 2, Table 1, and table S3). Of these, mammals comprised 12 taxa, fish consisted of 21 taxa, birds 6 taxa, and amphibians 1 taxon. A total of six taxa, including three small mammals, two fish, and one bird, were identified using both methods (Table 1). By contrast, 6 taxa, including, for example, tundra vole (*Alexandromys oeconomus*) and gray gurnard (*Eutrigla gurnardus*), were exclusively identified by osteological methods, while 28 taxa, such as the Norwegian lemming (*Lemmus lemmus*), hazel grouse (*Tetrastes bonasia*), and a range of fish species, were exclusively detected by aDNA BBM (Fig. 2 and Table 1).

### Reconstructing past faunal communities

The three distinct sediment layers at Nygrotta cover three separate periods from the Late Glacial–Holocene transition to the Middle Holocene. Bones were not equally distributed over the different layers. The oldest deposit (layer C) contained 4% (97 fragments) of the assemblage. These were almost exclusively fish bones, with the exception of three small mammal bones of the family Arvicolinae (two) and genus *Microtus* (one) (Table 1 and table S3). These mammal bones were recovered from the upper part of layer C close to the boundary with layer B. As layer C is otherwise exclusively marine, it is possible that these mammal bones are intrusive from the layer above and do not represent the faunal community present in layer C. The fish species in layer C are from six families representing at least three genera. Among the species identified are Arctic–boreal-related taxa like *Gaidropsarus* sp. (rocklings; which we adjust to *Gaidropsarus argentatus* Arctic rockling; table S6), Atlantic cod (*Gadus morhua*), common ling (*Molva molva*), and the families Pleuronectidae and Cottidae.

**Fig. 3.**
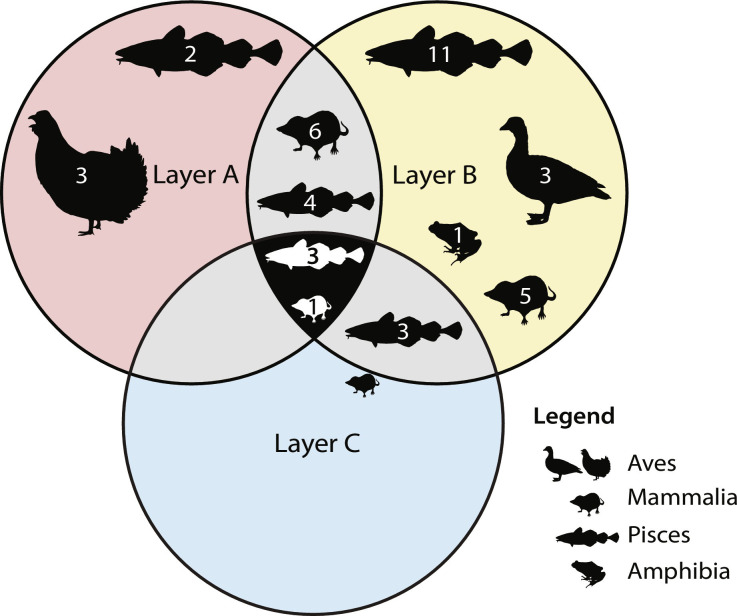
Comparison of identified taxa (BBM and osteology combined) per stratigraphic layers (A, B, and C) for Nygrotta. Venn diagram presenting the overlap and distinction of identified taxa based on the presence/absence in each of the three stratigraphic layers (A, B, and C). Silhouettes represent the taxonomic groups Aves, Mammalia, Pisces, and Amphibia. Numbers in silhouettes present the number of taxa.

The Early Holocene deposits (layer B) contained 90% of the faunal assemblage with 2143 bone fragments (table S3). These bones comprised seven mammalian families, with 10 genera and 9 species (Table 1), including large mammal genera such as *Ursus* (bears) and *Lepus* (hares and jackrabbits). The *Ursus* sp. was identified by osteology and must represent the brown bear (*Ursus arctos*) fitting the boreal assemblage at Nygrotta during the Early Holocene. Likewise, the *Lepus* sp. sequences were adjusted to mountain hare (*L. timidus*; table S6), the only *Lepus* species present in Fennoscandia. In addition to these large mammals, we also identified the genus *Canis* (wolves and dogs) and retrieved a high number of DNA reads assigned to Felidae (cats). The DNA marker used for BBM does not distinguish between wolf (*Canis lupus*) and domestic dog (*Canis lupus familiaris*), and the Felidae sequence has a 100% match to domestic cat (*Felis catus*), African wildcat (*Felis lybica*), and European wildcat (*Felis silvestris*). The Felidae identification was accordingly adjusted to the genus *Felis* (table S6). Last, we identified several small mammal species, including voles such as the short-tailed field vole (*Microtus agrestis*), Norwegian lemming (*L. lemmus*), and shrews like the common shrew (*Sorex araneus*). *L. lemmus* and *Sorex* sp. are present from the lowermost part of layer B and appear in the sequence before other small mammals (tables S5 and S7). The only birds identified in layer B are typically waterbirds such as Atlantic puffin (*Fratercula arctica*), gulls (Laridae), and the Anatidae family represented by the genus *Anser*. Fish were composed of 14 families, representing 15 genera and 14 species (Table 1). Although a small number of freshwater fish were detected by aDNA, including gudgeon (*Gobio gobio*) and stone loaches (*Barbatula* sp.), most species are representative of a marine habitat. These species include boreal indicator species such as saithe (*Pollachius virens*), cusk (*Brosme brosme*), and gray gurnard (*E. gurnardus*), as well as tidal or inshore species such as long-spined bullhead (*Taurulus bubalis*) and rock gunnel (*Pholis gunnellus*). Following both the number of identified specimens (NISP) and aDNA reads, the Gadidae, Pleuronectidae, and common ling (*M. molva*) are dominant in layer B (tables S3 and S5). All fish taxa identified in layer C were also found in layer B (Fig. 3). Frog DNA was detected in layer B, although the sequences could not be identified further than Ranidae.

The Middle Holocene deposit (layer A) contained 6% of the bone assemblage with 141 fragments. These bones comprised three mammalian families, six genera, and five species (Table 1 and table S3). *Felis* sp. is the only large mammal represented in layer A with the remainder consisting of small mammals. BBM identified the same voles and shrews in layer A as in layer B, but no lemming DNA was detected. Birds in layer A are from the Phasianidae family represented by black grouse (*Lyrurus tetrix*), capercaillie (*Tetrao urogallus*), and hazel grouse (*Tetrastes bonasia*). Fish were represented by seven families, seven genera, and six species (Table 1). Two of these fish taxa (*Esox lucius* and *Seriola* sp.) were not detected in the lower layers B and C (Fig. 3 and Table 1). By contrast, the freshwater species identified in layer B were not found in layer A. Boreal marine species continue into this phase with the presence of saithe (*P. virens*) and common dab (*Limanda limanda*). A number of cold-adapted species present in layer B are not detected in layer A, including Arctic rockling (*G. argentatus*) and lumpfish (*Cyclopterus lumpus*). In addition, certain boreal species such as common ling (*M. molva*) and cusk (*B. brosme*) that were identified in earlier layers were not found here, while the temperate amberjack genus *Seriola* emerges in layer A.

## DISCUSSION

Combining morphological analyses and BBM we identified the most diverse and complete faunal sequence from above the Arctic Circle in Fennoscandia, dated to three separate episodes between 13–5.6 ka cal B.P. The Nygrotta site shows a shallowing upward sequence (with hiatus) from shallow marine to tidal to subaerial habitat in response to the glacioisostatic rebound. Our high-resolution record gives insights into past faunal communities and individual species distributions during a period of strong climatic turnover. First, we obtain an early sequence of borealization above the Arctic Circle in both the marine and terrestrial environments following the Last Glacial retreat and into the HTM. Second, we confirm the presence of a number of species thought to have occupied the region but that have not previously been identified in the fossil record. Third, we identify species with expanded northern ranges, far outside their current geographic distribution. Notably, several of these observations, especially those within the marine environment, have clear parallels to current shifts in species distributions as a response to climate warming (*42*–*44*). Below, we discuss the biological and climatic implications of the animals identified in each of the recognized time periods (layers) and the entire Nygrotta assemblage.

Layer C (13,069–12,744 cal B.P.) coincides with the Late Glacial interstadial (14.0–12.9 ka), a period of mixed climatic conditions during which the Scandinavian Ice Sheet margins retreated rapidly and the relative sea level rose to ~90 m higher than present day (*35*, *45*). In the vicinity of Nygrotta, the ice sheet had retreated deeper into the fjord, and exclusively marine-related species recovered from the sand deposit in layer C support its interpretation as shallow marine sand and Nygrotta being submerged at the time. Shortly after the deposition of layer C, the Scandinavian Ice Sheet readvanced again in the transition to and during the early part of the Younger Dryas stadial (*46*–*49*). The ice advanced to the mouth of the fjord, ~27 km northwest of Nygrotta [Tysfjord; (*49*, *50*)], with the coastal area ice-free or covered by smaller local glaciers and ice caps (*46*, *48*, *51*, *52*). The identified marine species from layer C are consistent with the low Arctic climatic conditions in the area at this time (*47*, *53*–*55*). For instance, *G. argentatus* has a distinct Arctic distribution. Other species also have wide distributions among boreal and Arctic environments, such as members of the families Pleuronectidae and Cottidae or specific species like *G. morhua* (Atlantic cod) and *M. molva* [common ling; (*56*)]. A similar fish community to what we identify in Nygrotta (except for *G. argentatus*) was found in Blomvåg [~12.5 ka B.P.; (*26*)], the only other analyzed contemporary site that is located on the southern Norwegian coast about 1000 km south of Nygrotta. Overall, these fish communities from the Late Glacial–Holocene transition suggest a slightly colder adapted fauna lacking the warmer boreal species that are also observed today (*57*). These observations support estimates that sea temperatures during the Younger Dryas were colder than current temperatures (*58*).

Layer B (9534–9006 cal B.P.) coincides with the Early Holocene, a period with gradually increased warming, reaching temperatures higher than today after approximately 9 ka cal B.P. (*59*, *60*), but with intermittent episodes of cooling (*51*). Because of land uplift, relative sea levels dropped considerably in the Early Holocene from ~90 m above sea level (m.a.s.l.) and continued to drop more gradually up to the present day (*35*, *45*, *61*). In Nygrotta this is reflected in the high abundance of mussel shells and the appearance of terrestrial fauna in layer B, placing the 9.5–9 ka cal B.P. sea level at around 57 m.a.s.l. The sediment deposit forming layer B is interpreted as a partially tidal deposit, whereby inflowing water has brought sands and weathered local rock near the cave entrance further into the cave. The accumulation of marine and terrestrial faunal material is likely a combination of tidal flooding trapping marine life together with the build-up of predator and prey remains. The fish species observed in layer C are all present in layer B. In addition, we record ten species that currently have more boreal distributions, such as saithe (*P. virens*) and cusk (*B. brosme*). Several freshwater fish (*G. gobio* and *Barbatula* sp.) are also identified, which would present early colonizers following the glacial retreat. Different colonization routes for freshwater fish into northern Norway have been suggested. For instance, fish may have migrated following ice-fringe lakes from the east, driven by the high amount of meltwater from the glaciers as suggested for European whitefish [*Coregonus lavaretus*; (*62*)]. In addition, fish may have migrated through the Swedish river systems from the Baltic Sea, as suggested for species such as European perch (*Perca fluviatilis*) and northern pike (*62*–*65*). Either of these scenarios is plausible for *G. gobio* and *Barbatula* sp., which currently have a northern distribution in Russia close to the White Sea as well as in the Baltic (*66*). Nevertheless, the presence of a mountain range directly to the east of Nygrotta would form a natural barrier; we therefore hypothesize that initial freshwater fish migrations following the glacial retreat would have occurred further north and from there moved south down to Nygrotta. A third route, up from the south following the ice retreat, is less likely as modern northern populations of freshwater fish have a greater genetic affinity with populations in the Baltic and the northeast than with populations in southern Norway (*63*–*65*). Overall, our observations of these early freshwater colonizers above the Arctic Circle highlight their fast expansion into these high latitudes during the Early Holocene.

The terrestrial fauna of layer B shows a similar pattern as the marine fauna with a number of early boreal colonizers (mountain hare *L. timidus*, tundra vole *A. oeconomus*, Norwegian lemming *L. lemmus*, and shrews *Sorex* sp.) and later boreal migrants (e.g., bank vole *Clethrionomys glareolus* and *Microtus* sp.; table S7). A similar succession in Denmark shows an overlap of pioneer arctic with forest and taiga species (*24*). Several larger terrestrial boreal species (*Alces alces* and *Lynx lynx*) remain conspicuously absent from Nygrotta, yet we consider it likely that these were present in the region given that they were found at Sirijorda, a pit trap deposit ~270 km south of Nygrotta from a similar time period (*28*). Smaller mammals are well represented at Nygrotta and show a diversity analogous to that observed at Sirijorda (*28*), though several small mammal taxa are only observed in Sirijorda. For instance, the taiga shrew (*Sorex isodon*) and European water vole (*Arvicola terrestris*) were not found at Nygrotta. Conversely, the cold-adapted tundra vole (*A. oeconomus*) is found at Nygrotta but not present at Sirijorda (*28*). Overall, the small mammal diversity at Nygrotta reveals a boreal fauna with both early colonizers and later migrants, together indicative of grass/shrubland and forest habitat in the area (*67*–*69*).

The identification of *Canis* and *Felis* genera may represent remarkable findings of early wolf (*C. lupus*) and wildcat (*F. silvestris*) colonizers, yet need to be treated with caution as the genetic marker does not distinguish either of these from domestic dog and cat, respectively. With no evidence for human presence at the site and an Early Holocene age predating the earliest evidence, we currently have for *C. familiaris* in Norway [~8.5 ka B.P.; Vistehola (*31*)], the presence of domestic dogs at the site can be excluded. Likewise, the introduction of domestic cats (*F. catus*) into Fennoscandia dates to the Late Roman Iron Age (*70*), and in Norway, the earliest evidence comes from the Viking Age Oseberg ship burial [Archive University Museum of Bergen; (*71*)], postdating the Nygrotta deposits by at least 3500 years. Furthermore, Nygrotta was sealed until 1993, with the sedimentary deposits undisturbed and showing no signs of later intrusion. Nevertheless, domestic animals have long been recognized as contaminants in the laboratory, even though domestic cats and dogs are uncommon among such contaminants (*72*). In our study, the pattern of amplification of both *Canis* and *Felis* genera does not suggest that these stem from contamination. No sequences were assigned to *Canis* sp. or *Felis* sp. in any of the blanks nor in any amplifications of layer C. Moreover, *Canis* sp. was amplified in both repeats of a single sample only, and *Felis* sp. was amplified from five subsamples and, in all but one case, it was amplified in both repeats. Last, if either of these findings (*Canis* sp. or *Felis* sp.) would result from DNA leaching (*73*), then we would expect that the amount of contaminant DNA decreased with depth. We do not observe such a pattern, with *Felis* sp. being amplified in layers A and B2, while *Canis* sp. was detected in layer B3 only. DNA leaching therefore seems an unlikely explanation, but we nevertheless recognize that DNA leaching has only been studied as a source of contamination in sedimentary aDNA while it has to yet be investigated for bulk bone. If the Nygrotta samples represent wildcat (*F. silvestris*), then this would be remarkable, as this would be the highest latitude location for this species ever (*74*). The potential authentic identification of wildcats is an exciting prospect as remains across Europe are scarce (*74*, *75*). In Fennoscandia, the species has been recorded in Sjælland, Denmark, as part of the boreal fauna dated to 9.5 ka B.P. (*24*). In Norway, wildcats have only been found at three sites: Vistehola [~8.5 ka B.P.; (*31*)], Auve [(5–4 ka B.P.; (*76*)], and Årdal rock shelter (4–3 ka B.P.; Archive University Museum of Bergen)—all in southern Norway approximately 850–1000 km south of Nygrotta. The last evidence of wildcats in Fennoscandia comes from the site of Næsbyholm, Denmark, and dates to 2000 cal B.P. (*77*–*79*). The species became extinct in Fennoscandia following the end of the HTM, as temperatures dropped and snow cover increased above the wildcat threshold of 20 cm over a 100-day period (*75*, *80*). Estimates of past wildcat habitat limited their past distribution to the southwest coast of Norway (*81*), but our potential finding of wildcats at Nygrotta suggests that their range may have extended much further north already during the Early Holocene, at least along the coastline. Such early presence of wildcats would also be evidence for relatively low amounts of snow cover along Norwegian coastlines during this time and would suggest that the boreal pine and birch forests in Norway from 8 ka B.P. (*82*, *83*) provided suitable habitat for this species.

Layer A (5948–5611 cal B.P.) is interpreted as subaerial transported sand and coincides with the late HTM, locally dated from cores in the Lofoten Islands to ~7.5–5.5 ka cal B.P. in northern Norway (*84*). With estimates of around 1.5°–2.4°C warmer than 1961–1990 climate normals (*59*, *60*), the presence of warmer-adapted species during this period is expected. We observe the genus *Seriola* (Amberjacks) that has a current distribution limited to temperate and tropical waters (*85*). Past distributional shifts of warm water fish species have also been identified from faunal records in Sweden during the Late Holocene (*86*). Notably, this past northward shift of *Seriola* sp. is analogous to modern observations of *Seriola dumerili* (greater amberjack) moving north into the English Channel (*42*, *44*). Avifauna at Nygrotta during the late HTM show a switch from waterfowl to landfowl species such as black grouse (*L. tetrix*) and hazel grouse (*T. bonasia*). This transition may not only reflect the increased distance to water as the sea level continued to drop through the Holocene but also indicate the presence of nearby dense woodland habitats. Possible wildcat and the same small mammal community present in layer B are found in layer A, but we do not detect the early terrestrial colonizers such as hare and lemming that would be expected given their presence at contemporary sites in Fennoscandia (*28*, *87*) and suitable climatic conditions. Overall, the faunal record supports the warm climate that is known from the late HTM and provides evidence for species transitions and northward shifts that provide valuable comparative scenarios for responses to current climate warming.

The Nygrotta marine and terrestrial faunal chronology spans a long period of substantial climate warming above the Arctic Circle. There are no nearby records that allow direct comparison of the entire chronology because these records are constrained in their temporal range, have a more southern geographic location [e.g., Blomvåg, ~12.5 ka B.P. (*26*) and Skjonghelleren, 11.5–10 ka B.P. (*30*)], differ in the type of deposit [e.g., directly anthropogenically influenced such as Skipshelleren (*34*, *87*) and the Varangerfjord sites (*87*)], and/or differ in accumulation such as a forest pit trap [Sirijorda (*28*)]. The Nygrotta chronology therefore is the first to show a consistent change in faunal community structure in marine and terrestrial environments in response to substantial warming. First, we observe the appearance of warm adapted Amberjacks (*Seriola* sp.) coinciding with the HTM. Second, we see a similar pattern in the terrestrial environment with the appearance of more temperate-adapted species, such as the hazel grouse (*T. bonasia*) during the Early Holocene and HTM. Third, although we cannot affirm that species which are found in layer B are absent in layer A due to disparity in the bone count between both layers, we note that we did not observe cold-adapted species, such as *G. argentatus*, in layer A that covers the HTM. This observation fits with the presence of warmer-adapted species (*Seriola* sp. and *T. bonasia*) in layer A but not in B, coinciding with a warming mid-Holocene climate. Fourth, the succession of freshwater fish shows the fast colonization of suitable habitats coinciding with the disappearance of the Scandinavian Ice Sheet. Last, the appearance of black grouse (*L. tetrix*) and hazel grouse (*T. bonasia*) indicates that the climate supported dense woodland habitat during the HTM. Together, we obtain convincing evidence for major shifts in marine and terrestrial fauna in the high Arctic during the Holocene, and the faunal record at Nygrotta thus shows borealization as a naturally recurring phenomenon in periods of warming. Although we do not have a continuous sequence in Nygrotta limiting a detailed analysis of the pace of borealization during this time, there are a number of taxa in the assemblage that suggest rapid tracking of suitable habitat when it became available following the melting of the ice sheets (e.g., *Seriola* sp., *G. gobio*, and *M. agrestis*). Such rapid expansion as a result of ongoing global warming has also been observed in marine species moving into Arctic waters (*88*, *89*), yet in terrestrial systems taxa are now impeded by human modification of landscapes resulting in slower pace tracking of shifting habitat (*7*).

The analyses of the faunal sequence identified at Nygrotta fill a chronological and geographical gap in our knowledge of the recolonization of the far northern region of Fennoscandia during a period of dramatic climatic turnover following the end of the Late Glacial period. Complementing osteology with aDNA BBM has allowed us to exploit the 1000s of morphologically unidentifiable bone fragments retrieved from the site, exemplifying the power of integrating paleogenetic approaches with traditional osteology for maximizing the information content and resolution that can be obtained from such records. Here, this has resulted in a high-resolution faunal sequence covering the time from the Late Glacial–Holocene transition up to the late HTM. Being warmer than today, the HTM is often considered comparable with near-future climate estimates (*1*, *8*), and the transition into this period may provide useful indicators for current and future species responses (*16*). Notably, contemporary faunal assemblages dated to the HTM in Fennoscandia are almost always heavily anthropogenically influenced (*31*, *32*, *34*, *87*), highlighting the importance of rare sites like Nygrotta (this study) and Sirijorda (*28*) where faunal responses are not masked by anthropogenic overprinting. These natural paleo-archives provide unique windows into past species and community turnover.

## MATERIALS AND METHODS

### Excavation of subfossil remains

The Storsteinhola karst cave system is located near Kjøpsvik village, municipality of Narvik on the edge of Tysfjord in Nordland County, northern Norway (Fig. 1). The complete cave system has an aggregate length of ~2.6 km and a maximum height of 40 m (*36*). In 2021, we excavated the entrance of Nygrotta (68°50′ N 16°22′ E), which currently sits around 57 m.a.s.l. The excavation followed standard archeological excavation techniques (*90*–*92*). A grid system was implemented and orientated across the sediments at Nygrotta with the *x* axis running north-south and the *y* axis running east-west. Each 1-m square was further divided into quadrants (NE, SE, SW, and NW). Here, we focus on faunal material recovered from the northwest quadrant of square 106X/201Y, with the faunal assemblage from the remaining three quadrants preserved for future research. Square 106X/201Y is located in the inner part of Nygrotta’s first atrium and excavated to a depth of 0.5 m. The sediments were approached following stratigraphical-mechanical principles in which mechanical layers of 10 cm within the stratigraphical layers were excavated. All excavated sediments were wet sieved through 4- and 2-mm mesh to maximize recovery of small fragments. Sieved sediments were dried and subsequently stored at 4°C to reduce post-excavation DNA degradation. Bone fragment recovery was conducted offsite, wearing protective equipment (face masks and gloves) to minimize DNA contamination and using magnification lamps.

### Radiocarbon dating and chronological framework

Seven samples were selected for radiocarbon dating at the National Laboratory for Age Determination, Norwegian University of Science and Technology, Trondheim (fig. S1 and table S1). Calibration of the dates was done using OxCal version 4.4.4 software (*93*). Six of the samples had a δ^13^C value which indicated they had been influenced by the marine reservoir effect. These specimens were calibrated using the marine calibration curve Marine20 (*94*) with an Δ*R* offset of −100 ± 37 (*95*) taken from Tromsø, Norway (the closest data point to the site). Sample DRY6 had a δ^13^C value indicating a terrestrial diet and was calibrated using the calibration curve IntCal20 for the Northern Hemisphere (*96*). The calibrated age ranges reported are at the 95.4% probability standard. Numerical ages for defined periods such as the Early Holocene are taken from the chronostratigraphic chart updated in April 2023 by the International Commission on Stratigraphy (*97*).

### Archeology

All excavations at Nygrotta were conducted using archeological methodology looking for signs of human remains, constructed features, hearths, artifacts, and fire-cracked rocks. Potential artifacts of quartz and quartzite that could have been processed or reworked by humans were collected and taken into the laboratory for further analyses, to identify signs of knapping. Furthermore, faunal remains were analyzed for traces of butchery, cutting marks, human gnawing, and any indication of domesticated species or patterns of exploitation that could indicate human presence. In addition, careful observations of sieved sediments for organics such as charcoal and charred seeds were carried out.

### Osteological analysis

The osteological analysis of the faunal assemblage was performed through comparative morphology, whereby the recovered bones were compared to modern bones of known species using the reference collection at the University Museum of Bergen. Species abundance was quantified based on the NISP. Wherever possible, fragments belonging to a single specimen were counted as one to prevent overrepresentation of species. Because of the small amount of material available and the rarity of a site this age, all bone elements were taken into account and included within the NISP counts (including vertebrae and phalanges). To avoid overrepresentation when calculating the number of taxa, we counted genus and species level identifications, while family level was counted only when there were no species or genus level identifications within that specific family. Taphonomic markers were recorded where present and followed the guidance outlined in (*98*). To minimize contamination, face masks and gloves were always used while handling bones.

### Bulk-bone metabarcoding

The bone fragments not identifiable by comparative morphology were sorted into Mammalia, Pisces, Aves, and unidentifiable vertebrates according to their sediment layer. One bulk sample was made for each group (Mammalia, Pisces, Aves, and Unidentified) per layer (A1, B1, B2, B3, and C1). Complete bulk samples were milled into ~1-mm^3^ fragments using a stainless steel mortar and pestle (*99*) and up to three subsamples of about 110 mg, when that amount was available, were taken for DNA extraction per taxonomic group and layer (table S8). Twenty bulk samples were milled, and with the addition of subsamples, DNA was extracted from a total of 47 samples (table S8). Predigestion and DNA extraction protocols are followed by Lord *et al*. (*100*). Briefly, samples were predigested for 30 min followed by overnight digestion at 55°C. Extracts were subsequently concentrated and purified before amplification with up to three primer pairs specific for mammals, fish, and birds, respectively [Mamp007; Fish16S; Aves12S; (*101*, *102*); see Supplementary Text for details]. All pre–polymerase chain reaction (PCR) laboratory work was carried out in the dedicated aDNA laboratory at University of Oslo following standard protocols to minimize contamination.

Data were processed using the OBITools package v.1.2.12 (https://pythonhosted.org/OBITools/index.html) (*103*) following this pipeline: https://pythonhosted.org/OBITools/wolves.html (see Supplementary Text). For taxonomic assignment, sequences were compared to reference libraries (using *ecotag*) where reference libraries were built for each of the different primer pairs by performing an in silico PCR with *ecoPCR* (*104*) on the European Molecular Biology Laboratory (February 2022, www.ebi.ac.uk/ena/browser/home) and the National Center for Biotechnology Information (NCBI) Taxonomy database (www.ncbi.nlm.nih.gov/taxonomy). Sequences were further filtered in R v.4.3.0 (www.r-project.org/) removing sequences with <95% identity for fish and birds and <98% for mammals, taxa with a total read count under 200, and PCR replicates with less than 100 reads total. Taxonomic identifications of replicate PCRs were merged as no significant differences were identified between PCR replicates (see Supplementary Text and table S9). The resulting taxa list was reviewed by taxonomists specialized in the respective groups, and the Nucleotide Basic Local Alignment Search Tool from NCBI (https://blast.ncbi.nlm.nih.gov/Blast.cgi) was used to further explore taxonomic identifications and adjust identifications where needed (table S6).

## Supplementary Materials

### This PDF file includes:

Supplementary Text Fig. S1 Tables S1 to S10 References
