# Supplementary material for: Ancient DNA and osteological analyses of a unique paleo-archive reveal Early Holocene faunal expansion into the Scandinavian Arctic
Source: Sci Adv. 2024 Mar 29;10(13):eadk3032. doi: 10.1126/sciadv.adk3032 (PMC10980262; doi:10.1126/sciadv.adk3032)
Supplement: Supplementary file 1 — Supplementary Text Fig. S1 Tables S1 to S10 References [file sciadv.adk3032_sm.pdf]

Science Advances
AAAS

Supplementary Materials for

Ancient DNA and osteological analyses of a unique paleo-archive reveal Early Holocene faunal expansion into the Scandinavian Arctic

Aurélie Boilard *et al.*

Corresponding author: Aurélie Boilard, aurelibo@ibv.uio.no; Samuel J. Walker, s.j.walker@ibv.uio.no; Sanne Boessenkool, sanne.boessenkool@ibv.uio.no

Sci. Adv. **10**, eadk3032 (2024)
DOI: 10.1126/sciadv.adk3032

This PDF file includes:

Supplementary Text
Fig. S1
Tables S1 to S10
References

Supplementary Text

1. Bulk-bone metabarcoding methodology

1.1 DNA extraction and amplification

Pre-digestion and DNA extraction protocols followed Lord et al. (*100*). Samples were pre-digested in 700 ųl extraction buffer (Urea 1M, EDTA 0.5M at pH8) with 15 ųl proteinase K (18 mg/ml) at 55ºC for 30 minutes. Samples were subsequently digested overnight on a nutator at 55ºC with fresh extraction buffer and proteinase K. Following digestion, 500 ųl of supernatant was concentrated to ~100 ųl using an Amicon Ultra-0.5 Centrifugal Filter Unit (30kDa MWCO), purified with a MinElute PCR Purification Kit (QIAGEN) and eluted in 100 ųl elution buffer (EB buffer). Primers targeted a region of the *16S rRNA* gene of the mitochondria for amplification in Mammals and Fish, and a *12S* region for birds. Mammalian DNA was amplified using the Mamp007 primers [5'-CGAGAAGACCCTATGGAGCT-3', 5'-CCGAGGTCRCCCCAACC-3'; (*102*)], fish DNA was amplified using the Fish16S primers (5'-TACCAAAAACATCGCCTCYTG-3', 5'-CATTTAAAGACAAGTGATTRCG-3'; this study, designed by Laura Epp) and bird DNA with the Aves12S primers [5'-GATTAGATACCCCACTATGC-3', 5'-GTTTTAAGCGTTTGTGCTCG-3'(*101*)]. Samples with unknown bone fragments were amplified with all three primer pairs. PCR reactions (25 ųl) contained 2 units of AmpliTaq Gold DNA Polymerase (Applied Biosystems), 15 mM (Tris-HCL), 150 mM (KCl), 10X amplification buffer, 0.2 mM of each dNTP, 2.5 mM $MgCl_2$, 0.8 ug BSA, 0.2 ųM of each primer and 5 ul of template DNA. For amplifications with Mamp007, two units of blocking primer MamP007_B_Hum1, 5′-GGAGCTTTAATTTATTAATGCAAACAGTACCC-3′ were added to reduce the amplification of human DNA (*102, 105*). Forward and reverse primers were tagged with a unique 8 or 9 bp barcode at the 5' end to allow for multiplexing, with each primer pair having the same tag. The list of tags used can be found in Taberlet et al. (*106*). PCR cycling conditions were as follows: 10 minutes at 95ºC, 40 cycles at 95ºC for 30s, annealing temperature for 30s, 72ºC for 1 minute, and a final extension of 10 minutes at 72ºC. Annealing temperature was 55ºC for the mammal and bird primers, and 50ºC for the fish primers. Negative controls were included for all DNA extractions and PCRs. All laboratory protocols up to PCRs were carried out in the dedicated ancient DNA laboratory at the University of Oslo following standard protocols to minimize contamination.

Amplified products were visualized on agarose gels (1.5%) and pooled according to the strength of the bands (three categories were recognized: strong, medium and weak). Pools were purified using the MinElute PCR Purification Kit (QIAGEN) and concentrations of pools were measured using the Qubit 2.0 dsDNA BR Assay Kit (Thermo Fisher). Pools were subsequently combined equimolarly, and libraries were built using the TruSeq DNA Nano library preparation kit and sequenced on the Illumina NovaSeq 6000 S4 (150 bp PE) at the Norwegian Sequencing Center.

1.2 Bioinformatic analyses

Data was processed using the OBITools package v.1.2.12 [https://pythonhosted.org/OBITools/index.html; (*99*)] following https://pythonhosted.org/OBITools/wolves.html. Forward and reverse reads were assembled using *illuminapairedend* and samples were assigned with *ngsfilter*. Subsequently, reads were discarded if their quality score was < 40, tags had < 100% match, primers had >3 mismatches, or read length was < 20bp. Unique reads were merged and *obiclean* was used to remove singletons and identify likely PCR and/or sequencing artefacts by applying a 5% threshold ratio to reclassify "internal" sequences to their corresponding "head". For taxonomic assignment sequences were compared to reference libraries using *ecotag*. Reference libraries were built for each of the different primer pairs

(Mamp007, Fish16S and Aves12S) by performing an *in-silico* PCR with *ecoPCR (104)* on the European Molecular Biology Laboratory (EMBL, February 2022, https://www.ebi.ac.uk/ena/browser/home) and the NCBI Taxonomy database (https://www.ncbi.nlm.nih.gov/taxonomy). A sequence of the Norwegian lemming *Lemmus lemmus* was manually added to the reference library Brown et al. (*107*).

Following analyses with *ecotag* sequences were filtered in R v.4.3.0 (https://www.r-project.org/) to minimize misidentification by removing sequences with < 95% identity for fish and birds and < 98% for mammals, taxa with a total read count under 200 and PCR replicates with less than 100 reads total. No reads were assigned to the common contaminants *Bos* sp. or *Gallus* sp., and following filtering no reads assigned to the genera *Homo*, *Ovis* or *Sus* remained in the dataset. Post-filtering, one extraction negative control had reads identified to the Leuciscinae fish family. One sample contained reads identified to Leuciscinae (261 reads), which were conservatively removed. Post-filtering no sequences remained in any of the other negative controls.

For comparison of replicate PCRs only samples where both PCR replicates contained reads after filtering and analyses were considered. This led to a total dataset of 22 samples for the Mamp007 primer, 4 samples for the Aves12S primer, and 35 samples for Fish16S. A presence/absence matrix of each PCR repeat for a given sample was generated. The number of extra taxa was calculated, and a mean and standard deviation generated per taxonomic group (mammals, birds, fish). Analyzing two PCR replicates allowed for the identification of an additional 0.49 ± 1.07 taxa in Aves12S, 1.68 ± 2.61 in Mamp007, and 1.75 ± 1.71 taxa in Fish16S. Considering the similarity between replicates we merged taxonomic identifications from both PCR replicates for further downstream analyses (Table S9).

We also analyzed the effect of extracting DNA from multiple subsamples from a single bulk-bone sample, specifically assessing the benefit of two or three subsamples instead of just one. A total dataset of 13 samples were used for analyzing the effect two subsamples versus one subsample, and 10 samples were used for analyzing the effect of analyzing three subsamples versus two subsamples as well three subsamples versus one subsample. To avoid any impact of PCR replicates into this test, only PCR1 was used in this analysis. We generated a presence/absence matrix as described above for the analyses of PCR replicates and calculated the average number of additional taxa that was detected when adding subsamples (Table S10). Analyzing two subsamples versus one identified on average 2.92 ± 3.59 more taxa. Analyzing an additional sample (i.e., three subsamples versus two subsamples) added on average 2.70 ± 3.16 more taxa (Table S10). Finally, analyzing three subsamples versus just one lead to the identification of 5.00 ± 4.24 more taxa. We conclude that adding subsamples leads to the identification of more taxa and may be beneficial when sufficient material is present. For the current study we merged identifications from different subsamples per layer.

1.3 Adjustments of taxonomic identification

The complete taxa list was reviewed by several taxonomic experts with a good understanding of Northern European fauna. Anne Karin Hufthammer, professor at the University Museum of Bergen, Liselotte M. Takken-Beijersbergen, senior engineer at the University Museum of Bergen, Thijs van Kolfschoten, professor emeritus in archeology at the University of Leiden, reviewed the mammals. Ingvar Byrkjedal, professor emeritus and Nicolas Straube, associate professor and curator of ichthyology from the Department of Natural History at the University Museum of Bergen reviewed the fish. Samuel J. Walker at the University of Oslo reviewed the birds. Sequences were thereafter assessed with NCBI Nucleotide BLAST tool as a complementary analysis for taxa that were considered unlikely or unexpected to be present. When multiple sequences were identified to the same taxon, the sequence with the highest identity and highest abundance was selected for analyses with BLAST. A subset of taxonomic identifications was adjusted based on the assessment of the

taxonomists and the BLAST results (Table S6). After any manual taxonomic adjustments, the taxa lists from aDNA BMM and osteological analyses were combined.

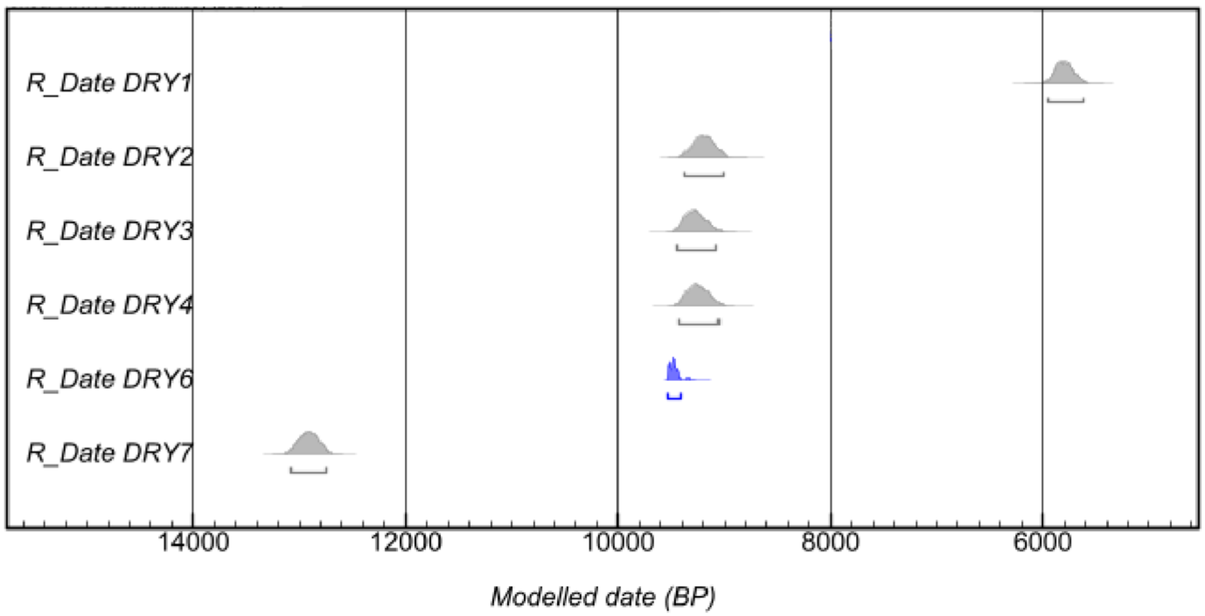

Figure S1. Modelled plot of radiocarbon dates for Nygrotta. Information on each sample, including the uncalibrated and calibrated dates are given in Table S1. Modelled using OxCal v.4.4.4 (*93*). DRY6, in blue, gave a terrestrial reading and was therefore modelled using the IntCal20 calibration curve for the Northern Hemisphere (*96*). All other dates were modelled on the Marine20 calibration curve (*94*) with a ΔR offset of -100 ± 37 (*95*).

Table S1. Radiocarbon dates for Nygrotta. Uncalibrated radiocarbon ages and calibrated ages at 95.4% probability. $\delta^{13}C$ values are given to justify marine or terrestrial models used to calibrate. BP = before present. Modelled using OxCal [Fig. S1; (*93*)], DRY6 gave a terrestrial reading and was therefore modelled using the IntCal20 calibration curve for the Northern Hemisphere (*96*). All other dates were modelled on the Marine20 calibration curve (*94*) with a ΔR offset of -100 ± 37 (*95*).

Specimen ID	Lab code	Layer	Sample	Radiocarbon Age (BP)	Calibrated date at 95.4% probability (BP)	$\delta^{13}C$
DRY1	Tra-17285	A	Shell	5515 ± 20	5948-5611 BP	-8.3
DRY2	Tra-17286	B1	Shell	8620 ± 30	9382-9006 BP	0.7
DRY3	Tra-17287	B3	Shell	8685 ± 30	9445-9084 BP	0.2
DRY4	Tra-17288	B3	Shell	8665 ± 25	9421-9062 BP	2.0
DRY5	Tra-17289	B3	Fish bone	Failed	Failed	Failed
DRY6	Tra-17290	B3	Bone	8445 ± 35	9534-9334 BP	-21.4
DRY7	Tra-17291	C1	Shell	11,490 ± 35	13,069-12,744 BP	2.9

Table S2. Bone element distribution by taxonomic group as identified by osteology. All fragments identified to bone element are included from all layers combined.

	Anser sp.	Unidentified Aves	Rodentia	Cricetidae/Arvicolinae	*Microtus* sp.	*Microtus agrestis*	*Alexandromys oeconomus*	*Clethrionomys glareolus*	*Sorex araneus*	*Sorex minutus*	*Ursus arctos*	Unidentified Mammalia	*Molva molva*	Gadidae	*Gadus morhua*	*Melanogrammus aeglefinus*	Labridae	*Eutrigla gurnardus*	Unidentified Pisces	**Total**
Cranium/maxilla/premaxilla	-	-	9	2	-	1	-	-	1	1	-	14	-	1	-	-	-	-	1	30
Mandible/angular/pharyngeum	-	-	-	-	-	2	2	-	3	1	-	-	2	3	-	-	-	-	3	16
Loose teeth/Dentary	-	-	20	6	7	12	1	1	-	1	-	-	2	-	-	-	2	-	49	101
Atlas	-	-	-	-	-	-	-	-	1	-	-	1	-	-	-	-	-	-	-	2
Axis	-	-	-	-	-	-	-	-	1	-	-	-	-	-	-	-	-	-	-	1
Vertebra	-	2	2	-	-	-	-	-	-	-	1	50	1	9	-	-	10	1	81	157
Sacrum	-	-	-	-	-	-	-	-	-	-	-	1	-	-	-	-	-	-	-	1
Scapula	-	-	1	-	-	-	-	-	-	-	-	2	-	-	-	-	-	-	-	3
Humerus	-	-	3	1	-	-	-	-	1	-	-	8	-	-	-	-	-	-	-	13
Radius	-	-	-	-	-	-	-	-	-	-	-	3	-	-	-	-	-	-	-	3
Ulna	-	-	1	-	-	-	-	-	1	-	-	2	-	-	-	-	-	-	-	4
Metapodial	-	-	1	-	-	-	-	-	-	-	-	12	-	-	-	-	-	-	-	13
Pelvis	-	-	8	-	-	-	-	-	3	-	-	10	-	-	-	-	-	-	-	21
Femur	-	-	7	-	-	-	-	-	-	-	-	17	-	-	-	-	-	-	-	24
Tibia	-	-	11	-	-	-	-	-	1	-	-	18	-	-	-	-	-	-	-	30
Calcaneus	-	-	-	-	-	-	-	-	-	-	-	3	-	-	-	-	-	-	-	3
Phalanx	1	-	-	-	-	-	-	-	-	-	-	1	-	-	-	-	-	-	-	2
Long bone	-	-	1	-	-	-	-	-	-	-	-	6	-	-	-	-	-	-	-	7
Otolith	-	-	-	-	-	-	-	-	-	-	-	-	-	-	2	1	-	-	-	3
Branchiostegal rays	-	-	-	-	-	-	-	-	-	-	-	-	-	9	-	-	-	-	12	21
Opercular/Opercular series	-	-	-	-	-	-	-	-	-	-	-	-	-	-	-	-	-	-	2	2
Pterygiophore	-	-	-	-	-	-	-	-	-	-	-	-	-	-	-	-	-	-	1	1
Lepidotrichia	-	-	-	-	-	-	-	-	-	-	-	-	-	-	-	-	-	-	5	5
Supracleithrum	-	-	-	-	-	-	-	-	-	-	-	-	-	-	-	-	-	-	1	1
Hyomandibula	-	-	-	-	-	-	-	-	-	-	-	-	-	-	-	-	-	-	2	2
Ceratohyal	-	-	-	-	-	-	-	-	-	-	-	-	-	-	-	-	-	-	1	1
Epihyale	-	-	-	-	-	-	-	-	-	-	-	-	1	-	1	-	-	-	1	3
Spina	-	-	-	-	-	-	-	-	-	-	-	-	-	-	-	-	-	-	16	16
**Total**	1	2	64	9	7	15	3	1	12	3	1	148	6	22	3	1	12	1	175	486

Table S3. Number of Identified Specimens (NISP) from Nygrotta per layer. All taxa identified by osteology and their corresponding NISP figure, which was calculated taking into account all bone elements (including vertebrae and phalanges). Unidentified fragments were identified to class (Aves, Mammalia and Pisces) where possible and into unidentified if class could not be determined. Ages are given in calibrated years before present (cal BP).

**ID**	**English name**	**A** 5948-5611 cal BP	**B** 9534-9006 cal BP	**C** 13,069-12,744 cal BP	**Total**
**Aves**					
Anser sp.	Grey & white geese	-	1	-	1
**Mammalia**					
Rodentia	Rodents	20	44	-	64
Cricetidae/Arvicolinae	Voles, lemmings and muskrats	3	4	2	9
Microtus sp.	Voles	3	3	1	7
Microtus agrestis	Short-tailed field vole	3	12	-	15
Alexandromys oeconomus	Tundra vole	2	1	-	3
Clethrionomys glareolus	Bank vole	-	1	-	1
Sorex araneus	Common shrew	-	12	-	12
Sorex minutus	Eurasian pygmy shrew	-	3	-	3
Ursus arctos	Brown bear	-	1	-	1
**Pisces**					
Molva molva	Common ling	-	6	-	6
Gadidae	Cods & haddocks	-	22	-	22
Gadus morhua	Atlantic cod	-	3	-	3
Melanogrammus aeglefinus	Haddock	-	1	-	1
Labridae	Wrasses	-	10	2	12
Eutrigla gurnardus	Grey gurnard	-	1	-	1
**Unidentified**					
Unidentified Aves		-	2	-	2
Unidentified Mammalia		86	151	4	241
Unidentified Pisces		17	1039	82	1138
Unidentified		7	826	6	839
**Total**		141	2143	97	2381

Table S4. Number of sequences and unique sequences remaining after each filtering step. Sequences were processed using the OBITools package v.1.2.12 [https://pythonhosted.org/OBITools/index.html (*103*), and filtered in R (version 4.3.0; R https://www.r-project.org/). *Part of the OBITools package.

**Filtering step**	**Program**	**Primer**	**Total reads**	**Unique sequences**
Raw reads from sequencing	-	-	50,443,140	-
Pairwise alignment	illuminapairedend*	-	50,443,140	-
Removal of sequences with <40 quality score	obigrep*	-	48,102,814	-
Assignment of sequences to samples	ngsfilter*	Aves12S	8,675,107	-
		Fish 16S	25,100,990	
		Mamp007	9,223,237	
Removal of reads with <20 bp and >165 bp length and merging identical reads	obigrep, obiuniq*	Aves12S	8,044,695	36,949
Removal of reads with <20 bp and >150 bp length and merging identical reads	obigrep, obiuniq*	Fish16S	23,151,009	348,099
		Mamp007	7,857,106	114,402
Matching the reference database	ecotag,obigrep*	Aves12S	8,044,695	36,949
		Fish16S	23,151,009	348,099
		Mamp007	7,857,106	114,402
Removal of PCR and sequencing errors	obiclean*	Aves12S	7,171,797	9386
		Fish16S	18,261,945	165,626
		Mamp007	6,751,802	54,764
Removal of sequences with <95% ID match	R	Aves12S	856	-
		Fish16S	59,752	
Removal of sequences with <98% ID match	-	Mamp007	566	-

Table S5. Number of reads per taxa identified by bulk-bone metabarcoding presented by stratigraphic layer (A, B, C). Original taxonomic identifications are presented, and identifications that were later manually adjusted or removed are marked with an asterisk (see Table S6).

ID	English name	A<br>5948-5611<br>cal BP	B<br>9534-9006<br>cal BP	C<br>13,069-12,744<br>cal BP
**Aves**				
Centrocercus sp.*	Sage grouse	2189	0	0
*Centrocercus urophasianus**	Greater sage-grouse	252	0	0
Dendragapus sp.*	Blue grouse	3734	0	0
Tympanuchus sp.*	Prairie chickens	1871	0	0
Tetraoninae	Grouse	1,647,321	0	0
Tetrao urogallus	Western capercaillie	1661	0	0
Lyrurus tetrix	Black grouse	1088	0	0
Tetrastes bonasia	Hazel grouse	315,094	0	0
Anatidae	Ducks, geese & waterfowl	0	298,822	0
Anser sp.	Grey & white geese	0	7655	0
Fratercula arctica	Atlantic puffin	0	98,350	0
Laridae	Gulls	0	1,109,264	
**Mammalia**				
Lepus sp.*	Hares	0	57,949	0
Microtus agrestis	Short-tailed field vole	507,915	110,322	0
Clethrionomys glareolus	Bank vole	41,142	110,488	0
Myodes rutilus	Northern red-backed vole	1983	1297	0
Lemmus lemmus	Norwegian lemming	0	59,099	0
Sorex sp.	Shrews	22,995	471,943	0
Sorex minutus	Eurasian pygmy shrew	2676	0	0
Felidae*	Cats	117,297	15,893	0
Phocidae	Earless seals	0	41,351	0
Canis sp.	Wolf/dog	0	1071	0
**Pisces**				
Gobio gobio	Gudgeon	0	683	0
Barbatula sp.	Stone loaches	0	288	0
Ipnops sp.*	Grideye	1386	455	0
Salmonidae	Salmon, trouts, chars etc.	0	698	0
*Esox masquinongy**	Muskellunge	2986	0	0
Molva molva	Common ling	0	5,523,655	574,487
Brosme brosme	Cusk	0	254,591	0
Gaidropsarus sp.*	Rockling	0	19,619	683
Gadidae	Cods & haddocks	226,601	7,475,735	1,111,650
Gadoidei	Subfamily of Gadidae	675	1,333,899	204,188
Gadus morhua	Atlantic cod	1690	101,430	17,786
Microgadus sp.*	Tomcod sp.	0	1203	0
Pollachius virens	Saithe	8505	32,321	0
Seriola sp.	Amberjacks	694	0	0
Pleuronectidae	Righteye flounders	57,298	740,763	11,608
Hippoglossus hippoglossus	Atlantic halibut	0	62,717	0
Limanda sp.*	Sole, flounders and dabs	189,829	31,914	0
Sebastidae	Rockfish & thornyheads	0	4645	0
*Sebastiscus marmoratus**	False kelpfish	0	1066	0
Cottidae	Sculpins	17,843	200,456	358,347
Taurulus bubalis	Long-spined bullhead	0	4045	0
*Artedius lateralis**	Smoothhead sculpin	1329	2299	2100
*Enophrys diceraus**	Antlered sculpin	0	1768	6146
Cyclopterinae	Lumpfishes	0	7015	0
Cyclopterus lumpus	Lumpfish	0	421	0
Pholis sp.	Gunnels	0	541	0
Pholis gunnellus	Rock gunnel	3744	13,239	0

Table S5 continued. Number of reads per taxa identified by bulk-bone metabarcoding presented by stratigraphic layer (A, B, C). Original taxonomic identifications are presented, and identifications that were later manually adjusted or removed are marked with an asterisk (see Table S6).

ID	English name	A<br>5948-5611<br>cal BP	B<br>9534-9006<br>cal BP	C<br>13,069-12,744<br>cal BP
Lycodinae	Eelpouts	0	211	0
*Anarrhichthys ocellatus**	Wolf eel	253	200	0
Anarhichas lupus	Atlantic wolffish	652	982	0
**Amphibia**				
Ranidae	True frogs	0	11,496	0
Microhylinae*	Subfamily of frogs	0	32,070	0
Nasikabatrachus sp.*	Purple frogs	0	315	0

Table S6. Manually adjusted or removed taxonomic identifications. Justifications for manual adjustment and removal of taxa identified from the bulk-bone metabarcoding data using the OBITools package v. 1.2.12 (*103*).

EcoTag ID	Adjusted ID	Justification
Centrocercus sp.	Phasianidae	Distributed in North America (*108*). We adjusted this identification to the family level of Phasianidae.
Centrocercus urophasianus	Phasianidae	Distributed in North America (*108*). We adjusted this identification to the family level of Phasianidae.
Dendragapus sp.	Phasianidae	Distributed in North America (*108*). We adjusted this identification to the family level of Phasianidae.
Tympanuchus sp.	Phasianidae	Distributed in North America (*108*). We adjusted this identification to the family level of Phasianidae.
Lepus sp.	*Lepus timidus*	Results of the NCBI-Blast gave a 100% match with five species of *Lepus*. As *Lepus timidus* (mountain hare) is the only species present in Fennoscandia, we shift this identification to species level.
Felidae	*Felis* sp.	Results of the NCBI-Blast gave a 100% match on both query cover and sequence identity with wildcat *Felis silvestris* (European wildcat), African wildcat (*Felis lybica*) and *Felis catus* (domestic cat), and no match with the native *Lynx lynx* (Eurasian Lynx) despite it being in the NCBI database. We adjust the identification to *Felis* sp.
Ipnops sp.	Removed	*Ipnops* sp. are deep sea fish and do not occur in the Norwegian Sea. The family Ipnopidae (deep-sea tripod fishes) are also not present in the Norwegian sea or surrounding waters. NCBI-Blast showed no 100% query cover matches. The sequence was amplified by primer pair Fish16S, does not match any common contaminants and its identification may instead reflect incomplete coverage of the reference database. Adjusting the taxonomic identification would lead to an identification beyond the family level. We removed this identification.
Esox masquinongy	*Esox lucius*	Native to North America (*109*). Results of NCBI-Blast were inconclusive, with no 100% query cover matches. However, as *Esox lucius* (northern pike) is the only *Esox* species in Fennoscandia we adjust the identification to this species.
Gaidropsarus sp.	*Gaidropsarus argentatus*	NCBI-Blast results gave a 100% match, on both query cover and 98.75% on sequence identity with both *Gaidropsarus argentatus* (Arctic rockling) and *Gaidropsarus ensis* (threadfin rockling). *Gaidropsarus ensis* only occurs in the western Atlantic (*110*) therefore, we adjust this identification to *G. argentatus*.
Microgadus sp.	Gadidae	There are two species in the *Microgadus* genus, *Microgadus proximus* (Pacific tomcod) and *Microgadus tomcod* (Atlantic tomcod). Neither of which occupy the Norwegian Sea. As a result, this identification was shifted to Gadidae.

Table S6 continued. Manually adjusted or removed taxonomic identifications. Justifications for manual adjustment and removal of taxa identified from the bulk-bone metabarcoding data using the OBITools package v. 1.2.12 Boyer et al.Boyer, Mercier, Bonin, Le Bras, Taberlet and Coissac (*103*).

EcoTag ID	Adjusted ID	Justification
Limanda sp.	*Limanda limanda*	Only one native species to Norway in the *Limanda* genus: *Limanda limanda* [common dab; ((*111*)]. NCBI-Blast results gave a 100% match on query cover and sequence identity with both *L. limanda* and *Limanda aspera* (yellowfin sole). Given the difference in their distribution [*L. aspera*; North Pacific; ((*111*))] we adjusted this identification to the species *L. limanda*.
Sebastiscus marmoratus	Sebastinae	Tropical species distributed in the Western Pacific. Interestingly one has been found in Norwegian waters (*112*) and this seems to be the only recording of the species in the North Atlantic. How it reached the North Atlantic is unknown but likely as larva or fry via a ballast water. Nevertheless, we adjusted the identification to the family of Sebastinae which has many taxa in the Atlantic.
Artedius lateralis	Cottidae	Native to the North Pacific (*113*). NCBI-Blast resulted in 13 matches at 100% query cover and 98.75% sequence identity, 10 of which are distributed in the North and East Pacific. Three are present in the Norwegian Sea: *Myoxocephalus Scorpius* (shorthorn sculpin), *Myoxocephalus quadricornis* (fourhorn sculpin) and *Gymnocanthus tricuspis* (Arctic staghorn sculpin). We adjusted this identification to the family level Cottidae.
Enophrys diceraus	Cottidae	Native to the North Pacific (*113*). NCBI-Blast results gave a 100% query cover and 98.75% sequence identity match with 16 species, the majority of which are distributed in the North and Northwest Pacific. Three species are currently found in the Norwegian Sea: *Gymnocanthus tricuspis* (Arctic staghorn sculpin), *Myoxocephalus Scorpius* and *Myoxocephalus quadricornis*. We adjusted this identification to the family level Cottidae.
Anarrhichthys ocellatus	*Anarhichas* sp.	Native to the North Pacific (*113*). NCBI-Blast results show a 100% query cover and 98.67% sequence identity match with two species native to Norway: *Anarhichas lupus* (Atlantic wolffish) and *Anarhichas minor* (spotted wolffish). *Anarrhichthys ocellatus* (wolf-eel) was also a 98.67% match, however it only occurs in the North Pacific (*113*). We adjusted this identification to the genus *Anarhichas.*
Microhylinae	Removed	Microhylinae are a subfamily of Mycrohylidae (narrow-mouthed frogs), few of which occur in the Northern Hemisphere and none of which occur in Europe. The sequence was amplified by primer pair Fish16S, it does not match any common contaminants and its identification may instead reflect incomplete coverage of the reference database. Adjusting the taxonomic identification would lead to an identification beyond the family level. This identification was removed.
Nasikabatrachus sp.	Removed	Endemic to India and the only genus in its family. NCBI Blast gave a match with 100% query cover and 97.26% sequence identity with two species endemic to the African continent (*Amnirana galamensis* and *Amnirana albolabris*). The sequence was amplified by primer pair Fish16S , it does not match any common contaminants and its identification may instead reflect incomplete coverage of the reference database. Adjusting the taxonomic identification would lead to an identification beyond the family level. This identification was removed.

Table S7. Taxa identified by BBM and osteology by stratigraphic and mechanical layer for layer B (B1, B2, B3). Layer A is dated to 5948-5611 cal BP, layers B1, B2 and B3 are dated to 9534-9006 cal BP and layer C is dated to 13,069 -12,744 cal BP.

ID	English name	A	B1	B2	B3	C
**Aves**						
Phasianidae	Pheasants, grouse & Allies	X	-	-	-	-
Tetraoninae	Grouse	X	-	-	-	-
Lyrurus tetrix	Black grouse	X	-	-	-	-
Tetrao urogallus	Western capercaillie	X	-	-	-	-
Tetrastes bonasia	Hazel grouse	X	-	-	-	-
Anatidae	Ducks, geese & waterfowl	-	-	-	X	-
Anser sp.	Grey & white geese	-	X	-	X	-
Fratercula arctica	Atlantic puffin	-	-	X	-	-
Laridae	Gulls	-	-	X	-	-
**Mammalia**						
Lepus timidus	Mountain hare	-	-	-	X	-
Cricetidae/Arvicolinae	Voles, lemmings and muskrats	X	-	X	-	X
Microtus sp.	Voles	X	X	X	-	X
Microtus agrestis	Short-tailed field vole	X	X	X	-	-
Alexandromys oeconomus	Tundra vole	X	X	-	-	-
Clethrionomys glareolus	Bank vole	X	X	X	X	-
Myodes rutilus	Northern red-backed vole	X	X	-	X	-
Lemmus lemmus	Norwegian lemming	-	X	X	X	-
Sorex sp.	Shrews	X	X	-	X	-
Sorex araneus	Common shrew	-	X	X	X	-
Sorex minutus	Eurasian pygmy shrew	X	-	X	X	-
Felis sp.	Wild/domestic cat	X	-	X	-	-
Phocidae	Earless seals	-	X	X	-	-
Ursus arctos	Brown bear	-	X	-	-	-
Canis sp.	Wolf/dog	-	-	-	X	-
**Pisces**						
Gobio gobio	Gudgeon	-	X	-	-	-
Barbatula sp.	Stone loaches	-	-	-	X	-
Salmonidae	Salmon, trouts, chars etc.	-	X	-	-	-
Esox lucius	Northern pike	X	-	-	-	-
Molva molva	Common ling	-	X	X	X	X
Brosme brosme	Cusk	-	-	X	-	-
Gaidropsarus argentatus	Arctic rockling	-	X	X	X	X
Gadidae	Cods & haddocks	X	X	X	X	X
Gadoidei	Subfamily of Gadidae	X	X	X	X	X
Gadus morhua	Atlantic cod	X	X	X	X	X
Melanogrammus aeglefinus	Haddock	-	-	X	-	-
Pollachius virens	Saithe	X	X	X	X	-
Seriola sp.	Amberjacks	X	-	-	-	-
Pleuronectidae	Righteye flounders	X	X	X	X	X
Hippoglossus hippoglossus	Atlantic halibut	-	-	X	X	-
Limanda limanda	Common dab	X	X	-	-	-
Labridae	Wrasses	-	-	X	-	X
Sebastidae	Rockfish & thornyheads	-	X	X	-	-
Eutrigla gurnardus	Grey gurnard	-	-	X	-	-
Cottidae	Sculpins	X	X	-	X	X
Taurulus bubalis	Long-spined bullhead	-	-	X	X	-
Cyclopterinae	Lumpfishes	-	-	-	X	-
Cyclopterus lumpus	Lumpfish	-	-	-	X	-
Pholis sp.	Gunnels	-	-	X	-	-
Pholis gunnellus	Rock gunnel	X	-	X	-	-
Lycodinae	Eelpouts	-	-	X	-	-
Anarhichas sp.	Wolffish	X	-	-	X	-
Anarhichas lupus	Atlantic wolffish	X	-	-	X	-
**Amphibia**						
Ranidae	True frogs	-	X	X	-	-

Table S8. Sample information of the 20 BBM samples analyzed. Layer presents the stratigraphic (letter) and mechanical (number) layer that the sample comes from. The number of subsamples from which DNA was extracted, the total weight of the available material and the total number of bone fragments in the total sample are given. DNA was extracted from up to three subsamples of about 110 mg where that amount was available from a total of 47 subsamples.

Class	BBM sample No.	Layer	No. of subsamples	Total weight of material (mg)	Total number of fragments
Pisces	BBY001	A1	1	90	17
Mammalia	BBY002	A1	2	300	42
Unidentified	BBY003	A1	3	600	72
Pisces	BBY004	B1	3	7550	93
Pisces	BBY005	B1	2	220	29
Mammalia	BBY006	B1	3	650	48
Unidentified	BBY007	B1	3	1010	114
Pisces	BBY008	B2	3	15,180	306
Pisces	BBY009	B2	3	3390	313
Mammalia	BBY010	B2	2	900	118
Unidentified	BBY011	B2	3	3563	250
Pisces	BBY012	B3	1	80	7
Pisces	BBY013	B3	3	3340	285
Mammalia	BBY014	B3	2	240	24
Unidentified	BBY015	B3	3	980	110
Unidentified	BBY016	B3	3	7930	280
Pisces	BBY017	C1	2	250	28
Pisces	BBY018	C1	3	600	55
Mammalia	BBY019	C1	1	100	4
Unidentified	BBY020	C1	1	8,6	6

Table S9. Average number of extra taxa detected by doing a second PCR replicate, per PCR primer. Only replicate pairs with reads remaining after filtering were considered. 22 samples were used for this analysis for the Mamp007 primer, 4 for Aves12S, and 35 samples for Fish16S.

	**Aves12S (n=4)**	**Fish16S (n=35)**	**Mamp007 (n=22)**
**Average**	1.75	0.49	1.68
**Standard deviation**	1.71	1.07	2.61

Table S10. The average number of extra taxa detected when extracting DNA from two or three subsamples. Only PCR1 was used for this test and only subsamples with reads post-filtering were included.

	Two versus one subsample (n=13)	Three versus two subsamples 3 (n=10)	Three versus one subsample (n=10)
**Average**	2.92	2.70	5.00
**Standard deviation**	3.59	3.16	4.24

REFERENCES

1. Intergovernmental Panel on Climate Change, "Climate Change 2023: Synthesis Report AR6. A Report of the Intergovernmental Panel on Climate Change" (Intergovernmental Panel on Climate Change, 2023).

2. M. Rantanen, A. Y. Karpechko, A. Lipponen, K. Nordling, O. Hyvärinen, K. Ruosteenoja, T. Vihma, A. Laaksonen, The Arctic has warmed nearly four times faster than the globe since 1979. *Commun. Earth Environ.* **3**, 168 (2022).

3. J. Melbourne-Thomas, A. Audzijonyte, M. J. Brasier, K. A. Cresswell, H. E. Fogarty, M. Haward, A. J. Hobday, H. L. Hunt, S. D. Ling, P. C. McCormack, T. Mustonen, J. A. Nye, M. Oellermann, R. Trebilco, I. van Putten, C. Villanueva, R. A. Watson, G. T. Pecl, Poleward bound: Adapting to climate-driven species redistribution. *Rev. Fish Biol. Fish* **32**, 231–251 (2022).

4. B. Elmhagen, D. Berteaux, R. M. Burgess, D. Ehrich, D. Gallant, H. Henttonen, R. A. Ims, S. T. Killengreen, J. Niemimaa, K. Norén, T. Ollila, A. Rodnikova, A. A. Sokolov, N. A. Sokolova, A. A. Stickney, A. Angerbjörn, Homage to Hersteinsson and Macdonald: Climate warming and resource subsidies cause red fox range expansion and Arctic fox decline. *Polar Res.* **36**, 3 (2017).

5. L. Nill, I. Grünberg, T. Ullmann, M. Gessner, J. Boike, P. Hostert, Arctic shrub expansion revealed by Landsat-derived multitemporal vegetation cover fractions in the Western Canadian Arctic. *Remote Sens. Environ.* **281**, 113228 (2022).

6. K. D. Tape, D. D. Gustine, R. W. Ruess, L. G. Adams, J. A. Clark, Range expansion of moose in Arctic Alaska linked to warming and increased shrub habitat. *PLOS ONE* **11**, e0152636 (2016).

7. J. Lenoir, R. Bertrand, L. Comte, L. Bourgeaud, T. Hattab, J. Murienne, G. Grenouillet, Species better track climate warming in the oceans than on land. *Nat. Ecol. Evol* **4**, 1044–1059 (2020).

8. K. D. Burke, J. W. Williams, M. A. Chandler, A. M. Haywood, D. J. Lunt, B. L. Otto-Bliesner, Pliocene and Eocene provide best analogs for near-future climates. *Proc. Natl. Acad. Sci. U.S.A.* **115**, 13288–13293 (2018).

9. C. Nolan, J. T. Overpeck, J. R. M. Allen, P. M. Anderson, J. L. Betancourt, H. A. Binney, S. Brewer, M. B. Bush, B. M. Chase, R. Cheddadi, M. Djamali, J. Dodson, M. E. Edwards, W. D. Gosling, S. Haberle, S. C. Hotchkiss, B. Huntley, S. J. Ivory, A. P. Kershaw, S.-H. Kim, C. Latorre, M. Leydet, A.-M. Lézine, K.-B. Liu, Y. Liu, A. V. Lozhkin, M. S. McGlone, R. A. Marchant, A. Momohara, P. I. Moreno, S. Müller, B. L. Otto-Bliesner, C. Shen, J. Stevenson, H. Takahara, P. E. Tarasov, J. Tipton, A. Vincens, C. Weng, Q. Xu, Z. Zheng, S. T. Jackson, Past and future global transformation of terrestrial ecosystems under climate change. *Science* **361**, 920–923 (2018).

10. J. C. Webb, A. E. Goodenough, Vegetation community changes in European woodlands amid a changing climate: A palaeoecological modelling perspective. *Community Ecol.* **22**, 319–330 (2021).

11. A. D. Barnosky, E. A. Hadly, P. Gonzalez, J. Head, P. D. Polly, A. M. Lawing, J. T. Eronen, D. D. Ackerly, K. Alex, E. Biber, J. Blois, J. Brashares, G. Ceballos, E. Davis, G. P. Dietl, R. Dirzo, H. Doremus, M. Fortelius, H. W. Greene, J. Hellmann, T. Hickler, S. T. Jackson, M. Kemp, P. L. Koch, C. Kremen, E. L. Lindsey, C. Looy, C. R. Marshall, C. Mendenhall, A. Mulch, A. M. Mychajliw, C. Nowak, U. Ramakrishnan, J. Schnitzler, K. D. Shrestha, K. Solari, L. Stegner, M A. Stegner, N. C. Stenseth, M. H. Wake, Z. Zhang, Merging paleobiology with conservation biology to guide the future of terrestrial ecosystems. *Science* **355**, eaah4787 (2017).

12. D. Nogués-Bravo, F. Rodríguez-Sánchez, L. Orsini, E. de Boer, R. Jansson, H. Morlon, D. A. Fordham, S. T. Jackson, Cracking the code of biodiversity responses to past climate change. *Trends Ecol. Evol.* **33**, 765–776 (2018).

13. T. P. Dawson, S. T. Jackson, J. I. House, I. C. Prentice, G. M. Mace, Beyond predictions: Biodiversity conservation in a changing climate. *Science* **332**, 53–58 (2011).

14. D. A. Fordham, H. R. Akçakaya, J. Alroy, F. Saltré, T. M. Wigley, B. W. Brook, Predicting and mitigating future biodiversity loss using long-term ecological proxies. *Nat. Clim. Change* **6**, 909–916 (2016).

15. M. C. Urban, G. Bocedi, A. P. Hendry, J.-B. Mihoub, G. Pe'er, A. Singer, J. R. Bridle, L. G. Crozier, L. De Meester, W. Godsoe, A. Gonzalez, J. J. Hellmann, R. D. Holt, A. Huth, K. Johst, C. B. Krug, P. W.

Leadley, S. C. F. Palmer, J. H. Pantel, A. Schmitz, P. A. Zollner, J. M. J. Travis, Improving the forecast for biodiversity under climate change. *Science* **353**, aad8466 (2016).

16. D. A. Fordham, S. T. Jackson, S. C. Brown, B. Huntley, B. W. Brook, D. Dahl-Jensen, M. T. P. Gilbert, B. L. Otto-Bliesner, A. Svensson, S. Theodoridis, J. M. Wilmshurst, J. C. Buettel, E. Canteri, M. M. Dowell, L. Orlando, J. A. Pilowsky, C. Rahbek, D. Nogues-Bravo, Using paleo-archives to safeguard biodiversity under climate change. *Science* **369**, eabc5654 (2020).

17. H. Renssen, H. Seppä, O. Heiri, D. M. Roche, H. Goosse, T. Fichefet, The spatial and temporal complexity of the Holocene thermal maximum. *Nat. Geosci.* **2**, 411–414 (2009).

18. H. Seppä, A. E. Bjune, R. J. Telford, H. J. B. Birks, S. Veski, Last nine-thousand years of temperature variability in Northern Europe. *Clim. Past* **5**, 523–535 (2009).

19. M. Widmann, Delayed Holocene warming. *Nat. Geosci.* **2**, 380–381 (2009).

20. A. L. Hughes, R. Gyllencreutz, Ø. S. Lohne, J. Mangerud, J. I. Svendsen, The last Eurasian ice sheets – a chronological database and time-slice reconstruction, DATED-1. *Boreas* **45**, 1–45 (2016).

21. S. Ekman, *Djurvärldens Utbredningshistoria på Skandinaviska Halvon* (A. Bonniers, 1922).

22. J. Lepiksaar, The Holocene history of theriofauna in Fennoscandia and Baltic countries. *Striae* **24**, 51–70 (1986).

23. A. Hufthammer, The Weichselian (c. 115,000–10,000 B.P) vertebrate fauna of Norway. *Boll. Soc. Paleontol. Ital.* **40**, 201–208 (2001).

24. K. Aaris-Sørensen, Diversity and dynamics of the mammalian fauna in Denmark throughout the last glacial-interglacial cycle, 115-0 kyr BP. *Foss. Strat.* **57**, 1–59 (2009).

25. A. K. Hufthammer, Animal osteology in Norway, in *Old Bones, Osteoarchaeology in Norway: Yesterday, Today and Tomorrow*, B. J. Sellevold, Eds. (Novus forlag, 2014), pp. 53–71.

26. R. Lie, Animal bones from the Late Weichselian in Norway. *Fauna Norveg. Ser. A.* **7**, 41–46 (1986).

27. P. Ukkonen, The post-glacial history of the Finnish mammalian fauna. *Ann. Zool. Fenn.* **30**, 249–264 (1993).

28. E. Østbye, S. E. Lauritzen, D. Moe, K. Østbye, Vertebrate remains in Holocene limestone cave sediments: Faunal succession in the Sirijorda Cave, northern Norway. *Boreas* **35**, 142–158 (2006).

29. F. Thörn, P. Rödin-Mörch, M. Cortazar-Chinarro, A. Richter-Boix, A. Laurila, J. Höglund, The effects of drift and selection on latitudinal genetic variation in Scandinavian common toads (*Bufo bufo*) following postglacial recolonisation. *Heredity* **126**, 656–667 (2021).

30. E. Larsen, S. Gulliksen, S. E. Lauritzen, R. Lie, R. Løvlie, J. Mangerud, Cave stratigraphy in western Norway; multiple Weichselian glaciations and interstadial vertebrate fauna. *Boreas* **16**, 267–292 (1987).

31. M. Degerbøl, Det osteologiske materiale, in *Fangst-Boplassen i Vistehulen på Viste, Randaberg, Nord-Jæren* (Stavanger Museum, 1951), pp. 52–93.

32. J. Rosvold, R. Andersen, J. D. Linnell, A. K. Hufthammer, Cervids in a dynamic northern landscape: Holocene changes in the relative abundance of moose and red deer at the limits of their distributions. *Holocene* **23**, 1143–1150 (2013).

33. H. Olsen, "Skipshelleren: Osteologisk materiale," thesis, University Museum of Bergen, Bergen, Norway (1976).

34. K. L. Hjelle, A. K. Hufthammer, K. A. Bergsvik, Hesitant hunters: A review of the introduction of agriculture in western Norway. *Environ. Archaeol.* **11**, 147–170 (2006).

35. J. S. Nese, "Sedimentologisk utvikling av grottene i Kjøpsvik – med hovudvekt på Stronsteinsholesystemet," thesis, University of Bergen, Bergen, Norway (1996).

36. Å. Lauritsen, S. E. Lauritzen, Quaternary cave and landform development in the Tysfjord Region, north Norway. *Karst Waters Special Publication* **2**, 73–77 (1996).

37. A. C. Grealy, M. C. McDowell, P. Scofield, D. C. Murray, D. A. Fusco, J. Haile, G. J. Prideaux, M. Bunce, A critical evaluation of how ancient DNA bulk bone metabarcoding complements traditional morphological analysis of fossil assemblages. *Quat. Sci. Rev.* **128**, 37–47 (2015).

38. D. C. Murray, J. Haile, J. Dortch, N. E. White, D. Haouchar, M. I. Bellgard, R. J. Allcock, G. J. Prideaux, M. Bunce, Scrapheap challenge: A novel bulk-bone metabarcoding method to investigate ancient DNA in faunal assemblages. *Sci. Rep.* **3**, 3371 (2013).

39. M. Antonosyan, F. V. Seersholm, A. C. Grealy, M. Barham, D. Werndly, A. Margaryan, A. Cieślik, T. W. Stafford, M. E. Allentoft, M. Bunce, L. Yepiskoposyan, Ancient DNA shows high faunal diversity in the Lesser Caucasus during the Late Pleistocene. *Quat. Sci. Rev.* **219**, 102–111 (2019).

40. F. V. Seersholm, H. Harmsen, A. B. Gotfredsen, C. K. Madsen, J. F. Jensen, J. Hollesen, M. Meldgaard, M. Bunce, A. J. Hansen, Ancient DNA provides insights into 4,000 years of resource economy across Greenland. *Nat. Hum. Behav.* **6**, 1723–1730 (2022).

41. F. V. Seersholm, D. J. Werndly, A. Grealy, T. Johnson, E. M. Keenan Early, E. L. Lundelius Jr., B. Winsborough, G. E. Farr, R. Toomey, A. J. Hansen, B. Shapiro, M. R. Waters, G. M. Donald, A. Linderholm, T. W. Stafford Jr., M. Bunce, Rapid range shifts and megafaunal extinctions associated with late Pleistocene climate change. *Nat. Commun.* **11**, 2770 (2020).

42. P. J . P. Whitehead, M. L. Bauchot, J. C. Hureau, J. Nielsen, E. Tortonese, *Fishes of the North-Eastern Atlantic and the Mediterranean*; UNESCO: Paris, France, 1986; Volume 2.

43. E. S. Poloczanska, M. T. Burrows, C. J. Brown, J. García Molinos, B. S. Halpern, O. Hoegh-Guldberg, C. V. Kappel, P. J. Moore, A. J. Richardson, D. S. Schoeman, W. J. Sydeman, Responses of marine organisms to climate change across oceans. *Front. Mar. Sci.* **3**, 62 (2016).

44. F. M. Porteiro, T. T. Sutton, I. Byrkjedal, A. Orlov, M. P. Heino, G. M. M. Menezes, O. A. Bergstad, Fishes of the Northern Mid-Atlantic Ridge collected during the MAR-ECO Cruise in June–July 2004. An annotated checklist (Oceanography Faculty Report 102, 2017).

45. J. Møller, Geometric simulation and mapping of holocene relative sea-level changes in Northern Norway. *J. Coast. Res.* **5**, 403–417 (1989).

46. B. G. Andersen, J. Mangerud, R. Sørensen, A. Reite, H. Sveian, M. Thoresen, B. Bergstrøm, Younger Dryas ice-marginal deposits in Norway. *Quat. Int.* **28**, 147–169 (1995).

47. T. O. Vorren, L. Plassen, Deglaciation and palaeoclimate of the Andfjord-Vågsfjord area, North Norway. *Boreas* **31**, 97–125 (2002).

48. B. Bergstrøm, L. Olsen, H. Sveian, The Tromsø-Lyngen glacier readvance (early Younger Dryas) at Hinnøya-Ofotfjorden, northern Norway: A reassessment. *Norges geologiske undersøkelse Bull.* **445**, 73–88 (2005).

49. J. S. Laberg, R. S. Eilertsen, G. R. Salomonsen, Deglacial dynamics of the Vestfjorden–Trænadjupet palaeo-ice stream, northern Norway. *Boreas* **47**, 225–237 (2018).

50. K. R. Fløistad, J. S. Laberg, T. O. Vorren, Morphology of Younger Dryas subglacial and ice-proximal submarine landforms, inner Vestfjorden, northern Norway. *Boreas* **38**, 610–619 (2009).

51. J. Bakke, S. O. Dahl, Ø. Paasche, R. Løvlie, A. Nesje, Glacier fluctuations, equilibrium-line altitudes and palaeoclimate in Lyngen, northern Norway, during the Lateglacial and Holocene. *Holocene* **15**, 518–540 (2005).

52. H. L. Jansen, S. O. Dahl, H. Linge, J. Bakke, P. R. Nielsen, B. C. Kvisvik, Palaeoclimatic and regional implications of Older Dryas and Younger Dryas local glacier activity in the low-Arctic valley Finnkongdalen, Andøya, northern Norway. *Boreas* **52**, 168–193 (2023).

53. T. Alm, Øvre Æråsvatn-palynostratigraphy of a 22,000 to 10,000 BP lacustrine record on Andøya, northern Norway. *Boreas* **22**, 171–188 (1993).

54. I. Aarnes, A. E. Bjune, H. H. Birks, N. L. Balascio, J. Bakke, M. Blaauw, Vegetation responses to rapid climatic changes during the last deglaciation 13,500–8,000 years ago on southwest Andøya, arctic Norway. *Veg. Hist. Archaeobotany* **21**, 17–35 (2012).

55. I. G. Alsos, P. Sjögren, A. G. Brown, L. Gielly, M. K. F. Merkel, A. Paus, Y. Lammers, M. E. Edwards, T. Alm, M. Leng, T. Goslar, C. T. Langdon, J. Bakke, W. G. M. van der Bilt, Last Glacial Maximum environmental conditions at Andøya, northern Norway; evidence for a northern ice-edge ecological "hotspot". *Quat. Sci. Rev.* **239**, 106364 (2020).

56. J. S. Nelson, *Fishes of the World* (Wiley, ed. 4, 2006).

57. C. W. Mecklenburg, A. Lynghammar, E. Johannesen, I. Byrkjedal, J. S. Christiansen, A. V. Dolgov,O. Karamushko, T. A. Mecklenburg, P. R. Møller, D. Steinke, R. M. Wienerroither, "Marine fishes of the arctic region volume 1," *CAFF Monitoring Ser. Rep.* (no. 28)= 2018).

58. U. Hoff, T. L. Rasmussen, H. Meyer, N. Koç, J. Hansen, Palaeoceanographic reconstruction of surface-ocean changes in the southern Norwegian Sea for the last ~130,000 years based on diatoms and with comparison to foraminiferal records. *Palaeogeogr. Palaeoclimatol. Palaeoecol.* **524**, 150–165 (2019).

59. A. E. Bjune, H. Birks, H. Seppä, Holocene vegetation and climate history on a continental-oceanic transect in northern Fennoscandia based on pollen and plant macrofossils. *Boreas* **33**, 211–223 (2004).

60. A. E. Bjune, J. Bakke, A. Nesje, H. J. B. Birks, Holocene mean July temperature and winter precipitation in western Norvay inferred from palynological and glaciological lake-sediment proxies. *Holocene* **15**, 177–189 (2005).

61. M. Billah, "Holocene relative sea-level changes in Evenes, northern Norway University of Bergen," thesis, University of Bergen, Bergen, Norway (2020).

62. J. Økland, K. Økland, *Dyr og Planter: Innvandring og Geografisk Fordeling. Vann og Vassdrag 1–4* (Vigmostad & Bjørke, ed. 4, 1999).

63. H. Huitfeldt-Kaas, *Ferskvandsfiskenes Utbredelse og Indvandring i Norge, Med et tillæg om Krebsen* (Centraltrykkeriet, 1918).

64. T. Kontula, R. Väinölä, Postglacial colonization of Northern Europe by distinct phylogeographic lineages of the bullhead, *Cottus gobio*. *Mol. Ecol.* **10**, 1983–2002 (2001).

65. U. Refseth, C. L. Nesbø, J. E. Stacy, L. A. Vøllestad, E. Fjeld, A. K. Jakobsen, Genetic evidence for different migration routes of freshwater fish into Norway revealed by analysis of current perch (*Perca fluviatilis*) populations in Scandinavia. *Mol. Ecol.* **7**, 1015–1027 (1998).

66. M. Kottelat, J. Freyhof, *Handbook of European Freshwater Fishes* (Kottelat, Cornol and Freyhof, 2007).

67. A. Myllymäki, Interactions between the field vole Microtus agrestis and its microtine competitors in Central-Scandinavian populations. *Oikos* **29**, 570–580 (1977).

68. L. Hansson, Competition between rodents in successional stages of taiga forests: Microtus agrestis vs. Clethrionomys glareolus. *Oikos* **40**, 258–266 (1983).

69. K. S. Hoset, H. Steen, Relaxed competition during winter may explain the coexistence of two sympatric *Microtus* species. *Annales Zoologici Fennici* **44**, 415–424 (2007).

70. J. Bitz-Thorsen, A. B. Gotfredsen, Domestic cats (*Felis catus*) in Denmark have increased significantly in size since the Viking Age. *Danish J. Archaeol.* **7**, 241–254 (2018).

71. A. W. Brøgger, H. Falk, H. Schetelig, *Osebergfundet, Utgit av den norske stat, Bind 1* (Distribuert ved Universitetet oldsaksamling, 1917).

72. J. A. Leonard, O. Shanks, M. Hofreiter, E. Kreuz, L. Hodges, W. Ream, R. K. Wayne, R. C. Fleischer, Animal DNA in PCR reagents plagues ancient DNA research. *J. Archaeol. Sci.* **34**, 1361–1366 (2007).

73. J. Haile, R. Holdaway, K. Oliver, M. Bunce, M. T. P. Gilbert, R. Nielsen, K. Munch, S. Y. Ho, B. Shapiro, E. Willerslev, Ancient DNA chronology within sediment deposits: Are paleobiological reconstructions possible and is DNA leaching a factor? *Mol. Biol. Evol.* **24**, 982–989 (2007).

74. R. S. Sommer, J. J. Crees, Late Quaternary biogeography of small carnivores in Europe in *Small Carnivores: Evolution, Ecology, Behaviour, and Conservation* (Wiley, 2022), chap. 4, 79–91.

75. R. Sommer, N. Benecke, Late Pleistocene and Holocene development of the felid fauna (Felidae) of Europe: A review. *J. Zool.* **269**, 7–19 (2006).

76. S. Isakson, A.K. Hufthammer, S. Bakkevig, M.S. Thomsen, Auve II, Tekniske og naturvitenskapelige undersøkelser, *Norske Oldfunn* **17**, 43–58 (1997)

77. J. G. Damm, Vildkatten, "*Felis silvestris silvestris*, og dens historie og udbredelse i Skandinavien i den postglaciale tid," thesis, University of Copenhagen, Copenhagen, Denmark (2000).

78. T. Hatting, Husdyrene, in *Dagligliv i Danmarks Middelalder. En arkæologisk kulturhistorie*, E. Roesdahl Ed. (Aarhus Universitetsforlag, 2004), pp. 110–122.

79. U. Møhl, Dyreknogler fra Næsbyholm Storskov. En plads fra ældre romersk Jernalder, in *Oldtidsagre i Danmark - Sjælland, Møn og Lolland Falster*, J. A. S. Skrifter, Ed. (Aarhus Universitetsforlag, 2010), pp. 259–273.

80. R. Piechocki, *Die Wildkatze Die neue Brehm - Bücherei* (Ziemsen Verlag, 1990).

81. A. C. Kitchener, E. Rees, Modelling the dynamic biogeography of the wildcat: Implications for taxonomy and conservation. *J. Zool.* **279**, 144–155 (2009).

82. P. Sjögren, C. Damm, Holocene vegetation change in northernmost Fennoscandia and the impact on prehistoric foragers 12 000–2000 cal. aBP– a review. *Boreas* **48**, 20–35 (2019).

83. I. G. Alsos, D. P. Rijal, D. Ehrich, D. N. Karger, N. G. Yoccoz, P. D. Heintzman, A. G. Brown, Y. Lammers, L. Pellissier, T. Alm, K. A. Bråthen, Postglacial species arrival and diversity buildup of northern ecosystems took millennia. *Sci. Adv.* **8**, eabo7434 (2022).

84. N. L. Balascio, R. S. Bradley, Evaluating Holocene climate change in northern Norway using sediment records from two contrasting lake systems. *J. Paleolimnol.* **48**, 259–273 (2012).

85. J. R. Paxton, D. F. Hoese, G. R. Allen, J. E. Hanley, *Petromyzontidae to Carangidae*, vol. 7 of *Zoological Catalogue of Australia,* (Australian Government Publishing Service, 1989).

86. L. Jonsson, Bogas-*Boops boops* (Linnaeus, 1758)-from the Biscay to the North Sea in 2500 BC and 1980 AD. *Munibe Antropologia-Arkeologia* **57**, 441–444 (2005).

87. H. Olsen, *Varanger-Funnene IV, Osteologisk Materiale*, vol. VII (Universitetsforlaget, Tromsø Museums Skifter, 1967).

88. M. Fossheim, R. Primicerio, E. Johannesen, R. B. Ingvaldsen, M. M. Aschan, A. V. Dolgov, Recent warming leads to a rapid borealization of fish communities in the Arctic. *Nat. Clim. Change.* **5**, 673–677 (2015).

89. I. V. Polyakov, M. B. Alkire, B. A. Bluhm, K. A. Brown, E. C. Carmack, M. Chierici, S. L. Danielson, I. Ellingsen, E. A. Ershova, K. Gårdfeldt, R. B. Ingvaldsen, Borealization of the arctic ocean in response to anomalous advection from sub-arctic seas. *Front. Mar. Sci.* **7**, (2020).

90. T. R. Hester, H. J. Shafer, K. L. Feder, *Field Methods in Archaeology* (Routledge, 2016).

91. C. Renfrew, P. G. Bahn, *Archaeology: Theories, Methods, and Practice* (Thames and Hudson, ed. 8, 2020).

92. P. Peregrine, *Archaeological Research: A Brief Introduction* (Routledge, 2021).

93. C. Bronk Ramsey, Bayesian analysis of radiocarbon dates. *Radiocarbon* **51**, 337–360 (2009).

94. T. J. Heaton, P. Köhler, M. Butzin, E. Bard, R. W. Reimer, W. E. Austin, C. B. Ramsey, P. M. Grootes, K. A. Hughen, B. Kromer, P. J. Reimer, Marine20—The marine radiocarbon age calibration curve (0–55,000 cal BP). *Radiocarbon* **62**, 779–820 (2020).

95. J. Mangerud, S. Gulliksen, Apparent radiocarbon ages of recent marine shells from Norway, Spitsbergen, and Arctic Canada. *Quatern. Res.* **5**, 263–273 (1975).

96. P. J. Reimer, W. E. Austin, E. Bard, A. Bayliss, P. G. Blackwell, C. B. Ramsey, M. Butzin, H. Cheng, R. L. Edwards, M. Friedrich, P. M. Grootes, The IntCal20 Northern Hemisphere radiocarbon age calibration curve (0–55 cal kBP). *Radiocarbon* **62**, 725–757 (2020).

97. K. M. Cohen, S. C. Finney, P. L. Gibbard, J. X. Fan, The ICS international chronostratigraphic chart. *Episodes J. Int. Geosci.* **36**, 199–204 (2013).

98. Y. Fernandez-Jalvo, P. Andrews, *Atlas of Taphonomic Identifications: 1001+ Images of Fossil and Recent Mammal Bone Modification* (Springer, 2016).

99. A. T. Gondek, S. Boessenkool, B. Star, A stainless-steel mortar, pestle and sleeve design for the efficient fragmentation of ancient bone. *Bio Techniques* **64**, 266–269 (2018).

100. E. Lord, A. Marangoni, M. Baca, D. Popović, A. V. Goropashnaya, J. R. Stewart, M. V. Knul, Noiret, M. Germonpré, E. L. Jimenez, N. I. Abramson, Population dynamics and demographic history of Eurasian collared lemmings. *BMC Ecol. Evol.* **22**, 1–13 (2022).

101. L. S. Epp, S. Boessenkool, E. P. Bellemain, J. Haile, A. Esposito, T. Riaz, C. Erseus, V. I. Gusarov, M. E. Edwards, A. Johnsen, H. K. Stenøien, New environmental metabarcodes for analysing soil DNA: Potential for studying past and present ecosystems. *Mol. Ecol.* **21**, 1821–1833 (2012).

102. C. Giguet-Covex, J. Pansu, F. Arnaud, P. J. Rey, C. Griggo, L. Gielly, I. Domaizon, E. Coissac, F. David, P. Choler, J. Poulenard, Long livestock farming history and human landscape shaping revealed by lake sediment DNA. *Nat. Commun.* **5**, 3211 (2014).

103. F. Boyer, C. Mercier, A. Bonin, Y. Le Bras, P. Taberlet, E. Coissac, obitools: A unix-inspired software package for DNA metabarcoding. *Mol. Ecol. Resour.* **16**, 176–182 (2016)

104. G. F. Ficetola, E. Coissac, S. Zundel, T. Riaz, W. Shehzad, J. Bessière, P. Taberlet, F. Pompanon, An in silico approach for the evaluation of DNA barcodes. *BMC Genomics* **11**, 434–10 (2010).

105. S. Boessenkool, L. S. Epp, J. Haile, E. V. A. Bellemain, M. Edwards, E. Coissac, E. Willerslev, C. Brochmann, Blocking human contaminant DNA during PCR allows amplification of rare mammal species from sedimentary ancient DNA. *Mol. Ecol.* **21**, 1806–1815 (2012).

106. P. Taberlet, A. Bonin, L. Zinger, E. Coissac, *Environmental DNA: For Biodiversity Research and Monitoring.* (Oxford Univ. Press, 2018).

107. T. Brown, D. P. Rijal, P. D. Heintzman, C. L. Clarke, H. P. Blankholm, H. I. Høeg, Y. Lammers, K. A. Bråthen, M. Edwards, I. G. Alsos, Paleoeconomy more than demography determined prehistoric human impact in Arctic Norway. *PNAS nexus* **1**, pgac209 (2022).

108. D. W. Winkler, S. M. Billerman, I. J. Lovette, Pheasants, grouse and allies (Phasianidae) in *Birds of the World*, S. M. Billerman, B. K. Keeney, P. G. Rodewald, T. S. Schulenberg, Eds. (Cornell Lab of Ornithology, 2020).

109. E. J. Crossman, Taxonomy and distribution, in *Pike Biology and Exploration*, J. F. Craig, Ed. (Chapman and Hall, 1996), pp. 1–11.

110. A. N. Svetovidov, Review of the three-bearded rocklings of the genus *Gaidropsaurus* Rafinesque, 1810 (Gadidae) with description of a new species. *J. Ichthyol.* **26**, 114–135 (1986).

111. K. A. Vinnikov, R. C. Thomson, T. A. Munroe, Revised classification of the righteye flounders (Teleostei: Pleuronectidae) based on multilocus phylogeny with complete taxon sampling. *Mol. Phylogenet. Evol.* **125**, 147–162 (2018).

112. H. Hansen, E. Karlsbakk, Pacific false kelpfish, *Sebastiscus marmoratus* (Cuvier, 1829) (Scorpaeniformes, Sebastidae) found in Norwegian waters. *Bioinvasions Rec.* **7**, 73–78 (2018).

113. W. N. Eschmeyer, E. S. Herald, H. Hammann, A field guide to Pacific coast fishes in North America (Houghton Mifflin Harcourt, 1983).
